# Premorbid Use of Beta-Blockers or Angiotensin-Converting Enzyme Inhibitors/Angiotensin Receptor Blockers in Patients with Acute Ischemic Stroke

**DOI:** 10.1155/2023/7733857

**Published:** 2023-02-01

**Authors:** Yuanyuan Zeng, Kelin Nie, Kevin L. Wallace, Fangfang Li, Jing Zhang, Caizhen Li, Yingying Wang, Jiewen Zhang, Jian Wang, Chao Jiang

**Affiliations:** ^1^Department of Neurology, The Fifth Affiliated Hospital of Zhengzhou University, Zhengzhou 450052, China; ^2^College of Mathematical and Natural Sciences, University of Maryland, College Park, MD 20742, USA; ^3^Department of Neurology, People's Hospital of Zhengzhou University, Zhengzhou 450000, China; ^4^Department of Anatomy, School of Basic Medical Sciences, Zhengzhou University, Zhengzhou, Henan 450001, China

## Abstract

This study was designed to investigate the impact of the preexisting use of beta-blockers, angiotensin-converting enzyme inhibitors (ACEIs), or angiotensin receptor blockers (ARBs) on the cellular immune response in peripheral blood and the clinical outcomes of patients with acute ischemic stroke. We retrospectively collected clinical data from a cohort of 69 patients with premorbid beta-blockers and 56 patients with premorbid ACEIs/ARBs. Additionally, we selected a cohort of 107 patients with acute ischemic stroke to be the control of the same age and sex. We analyzed cellular immune parameters in peripheral blood 1 day after the appearance of symptoms, including the frequencies of circulating white blood cell subpopulations, the neutrophil-to-lymphocyte ratio (NLR), and the lymphocyte-to-monocyte ratio (LMR). We found that the count of lymphocytes and the lymphocyte-to-monocyte ratio were significantly higher in the peripheral blood of patients treated with beta-blockers before stroke than in matched controls. However, the premorbid use of ACEIs/ARBs did not considerably impact the circulating immune parameters listed above in patients with acute ischemic stroke. Furthermore, we found that premorbid use of beta-blockers or ACEIs/ARBs did not significantly change functional outcomes in patients 3 months after the onset of stroke. These results suggest that premorbid use of beta-blockers, but not ACEIs/ARBs, reversed lymphopenia associated with acute ischemic stroke. As cellular immune changes in peripheral blood could be an independent predictor of stroke prognosis, more large-scale studies are warranted to further verify the impact of premorbid use of beta-blockers or ACEIs/ARBs on the prognosis of patients with ischemic stroke. Our research is beneficial to understanding the mechanism of the systemic immune response induced by stroke and has the potential for a therapeutic strategy in stroke interventions and treatment.

## 1. Introduction

Stroke is the second leading cause of death and disability worldwide, causing a substantial economic burden on families and society [1–4]. Acute ischemic stroke is the most common type, accounting for 70%-80% of all strokes and can cause severe neurological deficits [1, 4, 5]. Among survivors, the ability to work is compromised in 70% of victims, and 30% need help with self-care [1, 4, 6]. Thrombolysis and recanalization therapy are the only two FDA-approved treatment approaches with proven clinical benefits, but they must be performed within a narrow therapeutic window. Furthermore, thrombolysis and recanalization therapy have strict eligibility criteria and bleeding complications [4]. Therefore, it is critical to explore new therapeutic approaches, including prestroke interventions for patients with acute ischemic stroke.

Inflammation is essential in the pathophysiological process of ischemic stroke [7–10]. Evidence has indicated that increased sympathetic activity induced by ischemic stroke may contribute to the poststroke alteration of the systemic immune system, including changes in the percentages or counts of circulating neutrophils, lymphocytes, and monocytes [10–14]. On the contrary, alterations in the cellular immune system affect the outcomes of patients with acute ischemic stroke [10, 15]. A previous study has reported that stroke-induced sympathetic nervous system hyperactivity increased hematopoietic stem and progenitor cell activity in the bone marrow, consequently increasing neutrophil and monocyte counts in peripheral blood [16]. Further research revealed that a higher neutrophil count and an increase in monocyte numbers were independently associated with an adverse prognosis [17, 18]. Furthermore, a higher neutrophil-to-lymphocyte ratio (NLR) and a lower lymphocyte-to-monocyte ratio (LMR) value can be associated with worse outcomes 3 months after ischemic stroke [19, 20]. These studies suggested that the systemic immune response regulated by sympathetic activity may serve as a potential therapeutic target for ischemic stroke.

Additionally, increased sympathetic activity is associated with worse outcomes in patients with ischemic stroke [10]. Beta-blockers could inhibit hyperactivation of the sympathetic system and reverse chronic heart failure-induced lymphopenia [21]. However, the influence of beta-blockers on the peripheral cellular immune system is unclear in patients with acute ischemic stroke nor is the impact of beta-blockers on the prognosis of patients with acute ischemic stroke.

The sympathetic nervous system can also regulate the activation of the renin-angiotensin-aldosterone system (RAS), including serum angiotensin II (ATII) levels [22]. The ATII-stimulated angiotensin II receptor is necessary for monocyte exit from the spleen, potentially promoting systemic inflammatory responses [23]. The study has shown that the use of angiotensin-converting enzyme inhibitors/angiotensin receptor blockers (ACEIs/ARBs) independently improved the prognosis and reduced mortality in patients with high-risk coronary heart disease [24]. However, few studies have investigated the influence of ACEIs/ARBs on the peripheral cell immune system and the prognosis of patients with acute ischemic stroke. Knowledge of the impact of beta-blockers or ACEIs/ARBs on systemic immune changes and the outcomes of patients with acute ischemic stroke will further our understanding of the biological importance of these changes in patients with acute ischemic stroke.

Prestroke intervention with aspirin, metformin, statins, and physical activity has been shown to potentially aid in the prevention or treatment of stroke [25–28]. Given the documented influence premorbid factors have on stroke, the primary objective of this study was to systematically evaluate the influence of premorbid use of beta-blockers or ACEIs/ARBs on the subpopulations of circulating white blood cells and the prognosis of patients with ischemic stroke. First, we retrospectively evaluated the circulating immunocyte counts of patients with premorbid use of beta-blockers and ACEIs/ARBs. Then, we assessed the association between these variables and clinical covariates on admission or the outcomes of patients on day 90 after acute ischemic stroke. This study will help us to understand the innate and adaptive immune response in the periphery after ischemic stroke and to identify potential therapeutic targets to facilitate translational research.

## 2. Materials and Methods

### 2.1. Study Population

**Figure 1:**
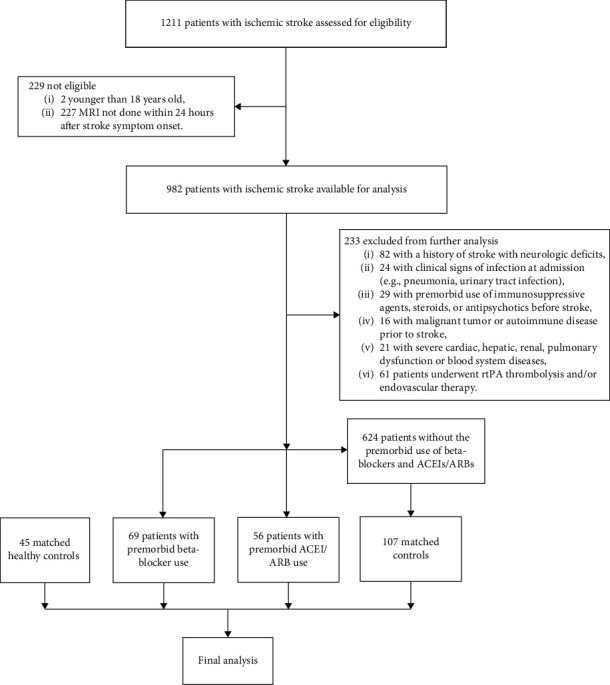
Flow chart of the study. rtPA: recombinant tissue plasminogen activator; ACEIs: angiotensin-converting enzyme inhibitors; ARBs: angiotensin receptor blockers.

This study was a retrospective analysis of the collected data from consecutive patients with acute ischemic stroke admitted to the Department of Neurology of the Fifth Affiliated Hospital of Zhengzhou University. The medical records of 1211 patients with acute ischemic stroke were reviewed at this academic center between January 2013 and December 2018. Patients were eligible if (1) they were older than 18 years, (2) had symptoms and signs of neurological deficits, (3) had a known time of symptom onset and were admitted to the hospital within 24 hours after stroke onset, (4) underwent magnetic resonance image (MRI) scan within 24 hours after stroke symptom onset, (5) met the requirements for the diagnosis of acute ischemic stroke [4]. Exclusion criteria were (1) a history of stroke with neurologic deficits, (2) clinical signs of infection at admission (e.g., pneumonia and urinary tract infection), (3) use of immunosuppressive agents, steroids, or antipsychotics before stroke, (4) malignant tumor or autoimmune disease before stroke, (5) severe cardiac, liver, renal, pulmonary dysfunction, or blood system diseases, (6) patients who underwent recombinant tissue plasminogen activator (rtPA) thrombolysis and/or endovascular therapy. Sixty-nine patients with premorbid use of beta-blockers and fifty-six patients with premorbid use of ACEIs/ARBs were recruited into this study. One hundred and seven patients with ischemic stroke without premorbid use of beta-blockers or ACEIs/ARBs were recruited as matched controls. Forty-five sex- and age-matched healthy subjects were recruited as healthy controls. All healthy controls came from the Health Checkup Center of the Fifth Affiliated Hospital of Zhengzhou University. Their health status was evaluated with a physical examination and routine laboratory tests. The exclusion criteria were the same as those described above. The flow chart for selecting patients in different groups is shown in Figure 1. The study protocol was approved by the Ethics Committee of the Fifth Affiliated Hospital of Zhengzhou University (K20180032). The study was carried out in accordance with the published International Health Guidelines (Declaration of Helsinki, 2008).

### 2.2. Clinical Data Collection

Baseline data and demographic data were collected, including age, sex, and a variety of risk factors including, but not limited to, hypertension, diabetes mellitus, coronary heart disease (CHD), hyperlipidemia, atrial fibrillation, smoking, alcohol use, and current use of medications such as statins and antiplatelet therapies. The clinical data collected included systolic blood pressure, diastolic blood pressure, a National Institutes of Health Stroke Scale score (NIHSS) at admission, and a modified Rankin Scale (mRS) score 90 days after the onset of symptoms. Laboratory panels were also collected and included general characteristics such as total cholesterol (TC), triglycerides (TG), high-density lipoprotein cholesterol (HDL-C), low-density lipoprotein cholesterol (LDL-C), and fasting blood glucose (FBG). Additional laboratory panels to measure the frequencies of circulating white blood cell subpopulations, including leukocyte, neutrophil, lymphocyte, monocyte, basophil, and eosinophil counts, measured as cells × 10^9^ per liter, were also retrospectively collected according to previous records. The neutrophil-to-lymphocyte ratio (NLR) was calculated as the neutrophil count divided by the lymphocyte count. The value of the lymphocyte-to-monocyte ratio (LMR) was calculated as the lymphocyte count divided by the monocyte count. The classification of ischemic stroke subtypes was based on the criteria of the Org 10172 Trial in Acute Stroke Treatment (TOAST) [29].

### 2.3. Measurement of Lesion Volume and Evaluation of Neurological Function

We gathered a panel of magnetic resonance images performed within 24 hours of stroke symptom onset using a standardized data collection form. All patients underwent a diffusion-weighted imaging examination, and a qualified neurologist determined the lesion volume. The infarct volume was calculated using the ABC/2 formula (A is the maximal longitudinal diameter, B is the maximal transverse diameter perpendicular to A, and C is the number of 5-mm slices containing the infarct) [30]. As previously described, the NIHSS was used to evaluate neurologic deficits in patients admitted to the neurology department [31]. This was followed by an mRS, a 7-point scale ranging from 0 (no symptoms) to 6 (death) [32], to assess neurologic outcomes 90 days after the onset of symptoms. To satisfy the proportional odds assumption, we reclassified the mRS scores as follows: mRS 0–3 (good outcome) and mRS 4–6 (poor outcome) [33, 34].

### 2.4. Statistical Analysis

Statistical analysis was performed with SPSS Statistics 13.0 software (SPSS Inc., Chicago, IL). Continuous variables were expressed as mean ± standard deviation or median (interquartile range). The Kolmogorov–Smirnov test was used to test the normality of the distribution. A Student's *t*-test or Mann–Whitney *U* test and one-way ANOVA or Kruskal-Wallis test followed by Bonferroni correction were appropriate for univariate analysis. Categorical variables were expressed as frequency or percentage, and differences between these variables were evaluated using the chi-square test or Fisher's exact test. Binary logistic regression was used to identify predictor variables for long-term outcomes. We performed a univariate analysis using appropriate tests for variables associated with the results on day 90. All associations significant at *p* < 0.10 were entered into a binary logistic model that adjusted for the correct variables. A value of *p* < 0.05 was considered statistically significant.

## 3. Results

### 3.1. Study Population

A total of 1211 consecutive patients with acute ischemic stroke were retrospectively screened. We enrolled 69 patients with premorbid beta-blocker use and 56 with premorbid ACEI/ARB use based on eligibility criteria. Other age-matched groups of 107 acute ischemic stroke patients and 45 healthy controls served as matched and healthy control groups, respectively. The beta-blocker cohort includes 43 men and 26 women, with an average age of 64.7 (±13.3) years. Patients in the beta-blocker cohort were more likely to have a history of arterial hypertension (*p* < 0.001), coronary artery disease (*p* < 0.001), atrial fibrillation (*p* < 0.001), diabetes mellitus (*p* = 0.001), previous stroke (*p* = 0.041), antiplatelet drug therapy (*p* < 0.001), and statin therapy (*p* < 0.001) than comparative matched controls. But serum levels of total cholesterol and low-density lipoprotein cholesterol in patients with premorbid beta-blocker use were significantly lower than in the matched controls (all *p* < 0.05). The patient group with premorbid use of ACEIs/ARBs consisted of 40 men and 16 women with a mean age of 63.0 (±11.8). Compared to the matched controls, more patients in the ACEI/ARB group have a history of arterial hypertension (*p* < 0.001), coronary artery disease (*p* = 0.005), diabetes mellitus (*p* < 0.001), atrial fibrillation (*p* = 0.037), or previous stroke (*p* = 0.005). The baseline characteristics of the matched controls, the beta-blocker group, and the ACEI/ARB group are shown in Table 1. A comparison of demographic characteristics and comorbidities between matched controls and healthy controls is presented in Supplementary Table 1. The doses of premorbid use of different beta-blockers and ACEIs/ARBs are summarized in Supplementary Table 2.

### 3.2. Impact of Beta-Blockers and ACEIs/ARBs on Immunocyte Subpopulations in Peripheral Blood from Patients with Acute Ischemic Stroke

Although there were no significant differences in the absolute count of leukocytes between the matched controls and healthy controls, total neutrophil count and NLR (all *p* < 0.05, Supplementary Table 1) were significantly higher in the matched controls than in healthy controls. We also found that lymphocyte count and LMR decreased significantly in matched controls compared to healthy controls (all *p* < 0.05, supplementary table 1). We then observed changes in the peripheral cellular immune system of the matched controls and patients with premorbid use of beta-blockers or ACEIs/ARBs. Compared to the matched controls, the premorbid use of beta-blockers only increased the absolute count of circulating lymphocytes and LMR (all *p* < 0.05, Table 1). However, we did not find differences in the frequencies of the immunocyte subpopulations between patients with premorbid ACEIs/ARBs and matched controls (all *p* > 0.05, Table 1).

### 3.3. Analysis of Immunocyte Subpopulations in Patients with Anterior Circulation Stroke (ACS) and Posterior Circulation Stroke (PCS)

**Figure 2:**
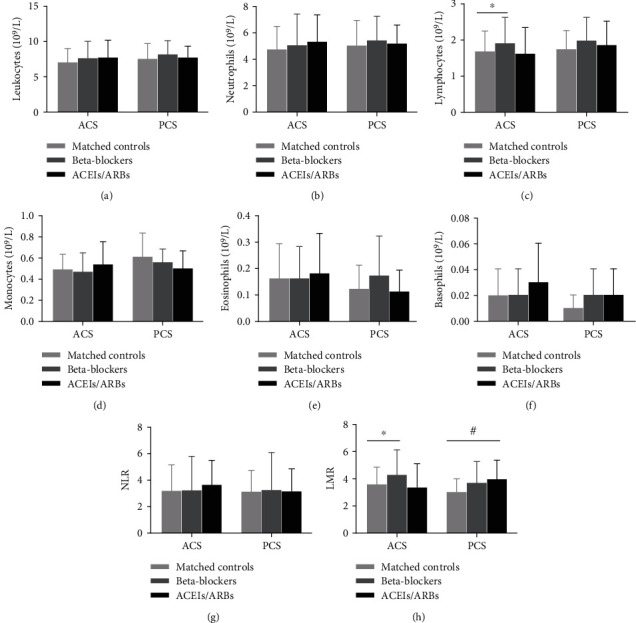
Analysis of peripheral immunocyte subpopulations in patients with anterior circulation stroke (ACS) and posterior circulation stroke (PCS). (a) Leukocyte counts, (b) neutrophil counts, (c) lymphocyte counts, (d) monocyte counts, (e) eosinophil counts, (f) basophil counts, (g) neutrophil-to-lymphocyte ratio (NLR), and (h) lymphocyte-to-monocyte ratio (LMR). ACS: matched controls, *n* = 81, beta-blockers, *n* = 52; ACEIs/ARBs, *n* = 37; PCS: matched controls, *n* = 26, beta-blockers, *n* = 17; ACEIs/ARBs, *n* = 19. One-way ANOVA followed by Bonferroni correction for the analysis of different immunocyte subpopulations. ^∗^*p* < 0.05, beta-blockers (ACS) versus matched controls. ^#^*p* < 0.05, ACEIs/ARBs (PCS) versus matched controls.

The frequency of lymphocytes in peripheral blood and LMR of patients with ACS with premorbid beta-blocker use was significantly higher than that of the matched controls (all *p* < 0.05, Figure 2). Likewise, the LMR of PCS patients with premorbid ACEIs/ARBs were significantly higher than that of the matched controls (*p* = 0.010, Figure 2). However, no differences in the immunocyte subpopulation counts of patients with ACS and PCS patients were found between patients with premorbid use of ACEIs/ARBs and matched controls (all *p* > 0.05, Figure 2). These results suggest that the location of the lesion may not have an influence on the frequencies of peripheral immunocyte subpopulations in patients with acute ischemic stroke.

### 3.4. Impact of Beta-Blockers and ACEIs/ARBs on Outcomes in Patients with Acute Ischemic Stroke

**Figure 3:**
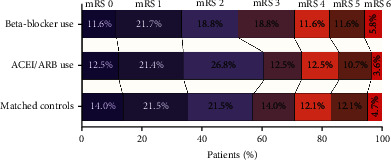
Distributions of the modified Rankin Scale (mRS) score in patients with ischemic stroke on day 90 after the onset of symptoms. Functional outcomes of matched controls (*n* = 107), patients with beta-blocker use before stroke (*n* = 69) and ACEIs/ARBs (*n* = 56) on day 90 after the onset of symptoms. The chi-square test was used for the analysis of functional outcomes. The significance did not differ between the 3 groups (all *p* > 0.05).

**Figure 4:**
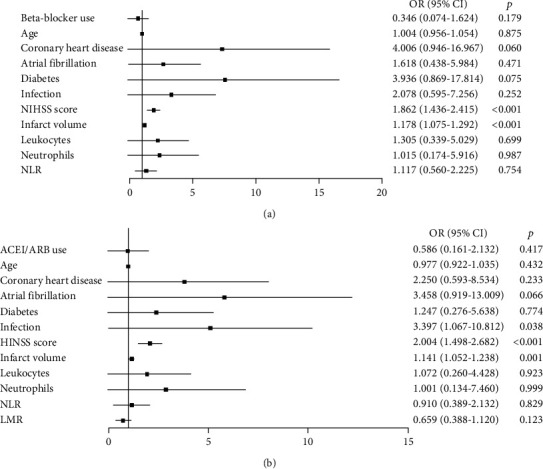
Binary logistic regression analysis of variables associated with functional outcomes on day 90 after ischemic stroke. The results showed that only a higher NIHSS score, a larger infarct volume at admission, and infection while in the hospital were independently associated with poor functional outcomes on day 90 after the onset of ischemic stroke (a, b). We did not find the influence of premorbid use of beta-blockers (a) or ACEIs/ARBs (b) on functional outcomes on day 90 after the onset of stroke.

The functional outcomes of patients with ischemic stroke 90 days after onset are shown in Figure 3. The differences were not significant between patients with premorbid use of beta-blockers or ACEIs/ARBs and the matched controls (Figure 3). Patients with premorbid beta-blockers and matched controls were dichotomized using the modified Rankin Scale (mRS) as having a favorable outcome (*mRS* = 0–3, *n* = 125) or an unfavorable outcome (*mRS* = 4–6, *n* = 51) 90 days after the onset of stroke. Univariate analysis revealed that patients with favorable outcomes were significantly younger than those with unfavorable outcomes (*p* = 0.002, Table 2). However, patients with unfavorable outcomes were more likely to have a history of coronary artery disease (*p* = 0.002, Table 2), atrial fibrillation (*p* = 0.004, Table 2), or diabetes mellitus (*p* = 0.002, Table 2). Additionally, the hospital infection rate, NIHSS score, and infarct volume of patients with favorable outcomes were significantly lower than those with unfavorable outcomes (all *p* < 0.001, Table 2). Furthermore, the counts of circulating leukocytes, neutrophils, and NLR were lower in patients with favorable outcomes than in patients with unfavorable outcomes (all *p* < 0.001, Table 2). Binary logistic analysis revealed that higher NIHSS scores on admission (adjusted odds ratio [OR] = 1.862; 95% confidence interval 1.436-2.415, *p* < 0.001; Figure 4(a)) and larger infarct volume on admission (adjusted OR = 1.178; 95% CI 1.075-1.291, *p* < 0.001; Figure 4(a)) were independently associated with poor functional outcomes on day 90 after the onset of stroke. However, we did not find a significant association between the premorbid beta-blocker use and functional outcomes of patients on day 90 after the onset of stroke (adjusted OR = 0.346; 95% CI 0.074-1.624, *p* = 0.179; Figure 4(a)).

Furthermore, patients with premorbid use of ACEIs/ARBs and matched controls were also dichotomized by the modified Rankin Scale (mRS) as having a favorable outcome (*n* = 117) or an unfavorable outcome (*n* = 46) on day 90 after ischemic stroke. Univariate analysis revealed that patients with favorable outcomes were also significantly younger than patients with unfavorable outcomes (*p* = 0.004, Table 3). Additionally, more patients had a history of atrial fibrillation (*p* = 0.006, Table 3) or diabetes mellitus (*p* = 0.042, Table 3) in patients with unfavorable outcomes than in patients with favorable outcomes. The infection rate, NIHSS score, and infarct volume of patients with favorable outcomes were significantly lower than those with unfavorable outcomes (*p* < 0.001, Table 3). Furthermore, leukocyte, neutrophil, and NLR counts were lower in patients with favorable outcomes than those with unfavorable outcomes (all *p* < 0.05, Table 3). However, the LMR was higher in patients with favorable outcomes than those with unfavorable outcomes (*p* = 0.016, Table 3). Binary logistic analysis also revealed that infection while in hospital (adjusted OR = 3.397; 95% CI 1.067-10.812, *p* = 0.038; Figure 4(b)), higher NIHSS scores at admission (adjusted OR = 2.004; 95% CI 1.498-2.682, *p* < 0.001; Figure 4(b)), and larger infarct volume (adjusted OR = 1.141; 95% CI 1.052-1.238, *p* = 0.001; Figure 4(b)) were independently associated with poor functional outcomes on day 90 after stroke onset. The premorbid use of ACEIs/ARBs had no impact on functional outcomes on day 90 after stroke (adjusted OR = 0.586; 95% CI 0.161-2.132, *p* = 0.417; Figure 4(b)).

## 4. Discussion

As in a previous study [35], we found notable changes in the cellular immune system of patients with acute ischemic stroke compared to healthy controls. Premorbid use of beta-blockers, but not ACEIs/ARBs, also significantly reversed changes in absolute lymphocyte count and LMR after acute ischemic stroke compared to matched controls. However, we did not determine how the location of the injury influences the overall effect on the cellular immune system in peripheral blood of patients with acute ischemic stroke. Furthermore, we did not find a significant correlation between the use of beta-blockers before stroke and ACEIs/ARBs on the clinical outcomes of patients with acute ischemic stroke on day 90 after ischemic stroke.

Ischemic stroke triggers a complex neuroinflammatory response involving changes in both immunocyte count and function, as well as an increase in proinflammatory cytokines, chemokines, proteases, and reactive oxygen species (ROS) [7, 36]. The local inflammatory response at sites injured by hypoxia-induced ischemia contributes to neuronal death, blood-brain barrier breakdown, hemorrhagic transformation, and neurologic deficits after ischemic stroke [2, 3, 37]. In addition to the activation of innate immunocytes, infiltrated macrophages, lymphocytes, and neutrophils in the peri-infarct region also participated in the local inflammatory response after ischemic stroke [7, 8, 36, 38]. Furthermore, circulating cells recruited to injured sites promoted local inflammatory events by increasing the production of cytokines such as interleukin (IL)-1*β*, IL-4, IL-6, and IL-10; tumor necrosis factor (TNF); interferons; monocyte chemotactic protein-1 (MCP-1) [8, 39]. The results indicate that infiltrating immunocytes into brain tissues is essential in the pathophysiology of ischemic stroke.

Previous studies have indicated that ischemic stroke causes profound changes in the peripheral blood [35]. These changes have been commonly found in patients with ischemic stroke and include pathologies such as a reduction in absolute lymphocyte count and increases in absolute neutrophil and monocyte counts [35]. As a stressor, ischemic stroke led to increased activation of the sympathetic nervous system and the renin-angiotensin-aldosterone system, including increased serum catecholamine and angiotensin II (Ang II) levels in patients with ischemic stroke [22, 40–44]. The beta-adrenergic receptor (*β*-AR) and the Ang II type 1 receptor (AT1) are commonly expressed by immunocytes and play a key role in driving these observed pathologies [45, 46]. Elevated catecholamines act on lymphocytes through *β*-AR inhibited T lymphocyte proliferation and promoted lymphocyte apoptosis by inducing an increase in the intracellular concentration of adenosine 3,5,-cyclic monophosphate (cAMP) and promoting activation of protein kinase A (PKA) [47, 48]. A previous study illustrated that using beta-receptor antagonists prevented the decrease in absolute lymphocyte counts in the peripheral blood of animals with ischemic stroke [49]. However, no study has investigated the influence of beta-receptor antagonists on cellular immunocytes in the peripheral blood of patients with acute ischemic stroke. Our study showed that the premorbid use of beta-blockers also increased the absolute count of lymphocytes in the peripheral blood of patients with ischemic stroke compared to matched controls. Changes induced by an ischemic stroke in the peripheral blood cell immune system can contribute to stroke-associated immunosuppression and increase the incidence of pneumonia or urinary tract infection [40, 50, 51]. Regulatory T cells (Treg) and alternatively activated macrophages (phenotype similar to M2) may exhibit immunosuppressive and neuroprotective effects after ischemic stroke [52, 53]. Activation of the sympathetic nervous system induced by ischemic stroke could reduce the frequency of circulating lymphocytes [10]. Studies also illustrated that catecholamines increased the frequency of circulating Treg cells and promoted the polarization of circulating macrophages to an alternatively activated M2-like (anti-inflammatory) phenotype in the acute phase of ischemic stroke [41, 54]. Future research on the influence of the use of beta-receptor antagonists on lymphocyte subpopulations and macrophage polarization in patients with ischemic stroke is warranted and may provide further insights.

The hypothalamic-pituitary-adrenal axis activation by ischemic stroke also increased cortisol secretion from the adrenal tract. Subsequently, it promoted apoptosis of B lymphocytes in peripheral blood [10, 41]. A previous study illustrated that using beta-blockers to block peripheral sympathetic nerves did not reverse the effects on the frequency of B lymphocytes in the peripheral blood of mice with transient middle cerebral artery occlusion (tMCAO). On the contrary, the frequency of B lymphocytes increased in the peripheral blood of mice lacking the glucocorticoid receptors CD19-Cre loxP Nr3c1 after tMCAO [55]. Catecholamines and cortisol increased remarkably after ischemic stroke [56]. Furthermore, B lymphocytes also account for a large proportion of lymphocytes. The contribution of the sympathetic nervous system to the peripheral immune system may warrant further study after an ischemic stroke.

Angiotensin II (Ang II), an essential component of the renin-angiotensin-aldosterone system, can also regulate the differentiation, activation, and chemotaxis of immunocytes, including macrophages, dendritic cells, T lymphocytes, and Th1 and Th17 cells [46]. Previous studies have illustrated that lisinopril, an ACEI inhibitor, protected the kidneys by alleviating macrophage infiltration and reduced MCP-1 expression in the renal interstitial of animals with progressive nephropathy [57]. Additionally, studies revealed that high doses of losartan (60 mg/kg) or perindopril (6 mg/kg) inhibited monocyte release from the spleen into peripheral blood [58]. Mechanism-wise, ACEIs/ARBs can inhibit immunocyte differentiation, chemotaxis, and infiltration by inhibiting proinflammatory cytokine secretion, diminishing the expression of adhesion molecules, and normalizing CRP concentration in blood plasma [59]. A separate previous study has also illustrated that the sympathetic nervous system can enhance the activation of the renin-angiotensin-aldosterone system under stress [60].

In contrast, the renin-angiotensin-aldosterone system could also increase serum catecholamines and cortisol levels by improving the activation of the sympathetic nervous system and the hypothalamic-pituitary-adrenal axis [61, 62]. It merges into the idea that the role of the renin-angiotensin-aldosterone system and the sympathetic nervous system in the immune response is contradictory in patients with cardiovascular disease. The sympathetic nervous system and the renin-angiotensin-aldosterone system have different effects on the immune response under stress. The sympathetic nervous system can reduce the frequency of circulating lymphocytes by inhibiting T lymphocyte proliferation and promoting lymphocyte apoptosis in patients with chronic heart failure or ischemic stroke [21, 48]. However, the renin-angiotensin-aldosterone system can increase the frequency of circulating monocytes by stimulating the release of monocytes from the spleen after myocardial infarction [21, 58]. Although ACEIs and ARBs can antagonize the activation of the renin-angiotensin-aldosterone system, no studies have observed the influence of ACEIs and ARBs on the peripheral immune system after acute ischemic stroke. In this study, we found that ACEIs/ARBs did not influence the systemic immune response of peripheral blood in patients with ischemic stroke.

Alternatively, acute stroke can influence the immune response by promoting shrinkage of the spleen volume via the activation of the sympathetic nervous system [10]. Furthermore, changes in the activation of the parasympathetic nervous system can also affect stroke outcomes by regulating the local and systemic immune response [10]. However, the efficacy of premorbid beta-blocker use on the prognosis of patients with ischemic stroke is currently controversial. An animal study revealed that beta-blockers decreased the incidence of spontaneous pneumonia after cerebral infarction [63]. A clinical study illustrated that atenolol alleviated systemic inflammatory response syndrome, inhibited the occurrence of pneumonia, and reduced mortality in patients with cerebral hemorrhage [64]. A large cohort retrospective analysis showed that using beta-blockers reduced mortality in stroke patients, suggesting that beta-blockers may influence the prognosis of patients with ischemic stroke [65]. Although these previous studies have shown promise, Maier et al. [66] found that beta-blockers did not reduce the incidence of poststroke pneumonia, sepsis, and death, while increasing the incidence of poststroke urinary tract infections. Additional clinical studies showed that premorbid beta-blockers did not influence stroke severity and neurologic function in patients with ischemic stroke [67, 68]. These findings are in line with our own study's findings, that when compared to a matched control group, the premorbid use of beta-blockers does not influence the functional outcomes of patients on day 90 after ischemic stroke. A previous study has revealed that catecholamines may regulate immune system activation through *β*2AR on immunocytes [47]. The use of *β*1AR blockers (e.g., metoprolol) in some patients may explain why the results of this study differ from previous studies.

Multiple trials have shown that antihypertensive drugs targeting the renin-angiotensin system significantly reduced the incidence of stroke [69, 70]. However, an animal study revealed that the infarct volume of Ang II-infused mice was greater than that of vehicle controls after tMCAO [71]. Furthermore, a significant reduction in infarct volume was also observed in mice with AT1R gene deficiency than in wide-type mice with tMCAO [71]. Moreover, an animal study revealed that premorbid use of ACEIs worsened neurologic functions after traumatic brain injury [72]. To date, no study has observed the influence of ACEIs/ARBs on the functional outcomes of patients with ischemic stroke. In this study, we found that the premorbid use of ACEIs/ARBs did not have an impact on the functional outcomes of patients with ischemic stroke on day 90. The effects of premorbid use of ACEIs/ARBs on stroke outcomes warrant further investigation.

Our study has limitations due to the small sample size. However, this small sample size may partially explain why premorbid use of beta-blockers only reversed changes in absolute lymphocyte count and LMR but did not affect clinical outcomes in patients with acute ischemic stroke. As this study is carried out retrospectively, prospective studies with larger sample sizes and extended follow-up periods are needed to validate our findings. Furthermore, stroke has a complex disease process, and the index of the absolute count of circulating lymphocytes and LMR in this study may not be sufficient to adequately evaluate the peripheral immune system. However, we limited our study to the effects of beta-blockers and ACEIs/ARBs on leukocyte subtypes in peripheral blood within 24 hours after stroke. We did not measure the cellular immune response at multiple time points.

The small sample size of this study imposes a limit on the further analysis of the influence of different AR blockers on the systemic immune response and outcomes of patients with ischemic stroke. In this study, we calculated infarct volume with the ABC/2 formula but not with more professional software like RAPID. In future research, we will study the influence of *β*2AR blockers, *β*1AR blockers, and nonselective beta-receptor blockers on the immune response and outcomes of patients with ischemic stroke. We will calculate the infarct volume with more professional software such as RAPID. We will also determine the association between changes in serum catecholamine or cortisol levels and the cellular immune system in peripheral blood from patients with ischemic stroke.

## 5. Conclusions

Our study suggested that the premorbid use of beta-blockers before stroke increased the frequency of lymphocytes and LMR in the peripheral blood of patients with acute ischemic stroke. However, we did not observe the impact of premorbid use on the prognosis of patients with ischemic stroke. In addition, we did not find a significant effect of the premorbid use of ACEIs/ARBs on peripheral immunocyte subpopulations or neurological functions of patients with ischemic stroke on day 90. Therefore, preclinical models should be used to determine the effects of beta-blockers or ACEIs/ARBs on the peripheral immune system and functional outcomes after ischemic stroke.

## Figures and Tables

**Table 1:** Baseline data for matched controls and patients with premorbid use of beta-blockers and ACEIs/ARBs.

Characteristic and variables	Matched controls (*n* = 107)	Beta-blockers (*n* = 69)	ACEIs/ARBs (*n* = 56)
Male, gender, *n* (%)	68 (63.6)	43 (62.3)	40 (71.4)
Age (years, mean ± SD)	61.4 ± 11.6	64.7 ± 13.3	63.0 ± 11.8
Hypertension, *n* (%)	64 (59.8)	64 (92.8)^∗∗∗^	56 (100.0)^†††^
Coronary heart disease, *n* (%)	19 (17.8)	40 (58.0)^∗∗∗^	21 (37.5)^††^
Atrial fibrillation, *n* (%)	19 (17.8)	30 (43.5)^∗∗∗^	18 (32.1)^†^
Diabetes, *n* (%)	11 (10.3)	20 (29.0)^∗∗^	19 (33.9)^†††^
Hyperlipidemia, *n* (%)	45 (42.1)	27 (39.1)	27 (48.2)
Previous stroke, *n* (%)	16 (15.0)	19 (27.5)^∗^	19 (33.9)^††^
Prior antiplatelets, *n* (%)	11 (10.3)	29 (42.0)^∗∗∗^	11 (19.6)
Prior statins, *n* (%)	10 (9.3)	25 (36.2)^∗∗∗^	9 (16.1)
Smoking, *n* (%)	49 (45.8)	25 (36.2)	24 (42.9)
Alcohol drinking, *n* (%)	37 (34.6)	23 (33.3)	21 (37.5)
Infection while in the hospital, *n* (%)	27 (25.2)	17 (24.6)	19 (33.9)
Systolic blood pressure at admission, mean ± SD, mmHg	155.2 ± 24.6	150.6 ± 20.6	152.6 ± 24.5
Diastolic blood pressure at admission, mean ± SD, mmHg	90.7 ± 14.8	88.3 ± 16.1	88.8 ± 13.6
Fasting blood glucose, mean ± SD, mmol/L	6.02 ± 2.10	5.95 ± 1.84	5.85 ± 1.96
Admission blood lipid			
TC, mean ± SD, mmol/L	4.48 ± 0.83	4.12 ± 0.98^∗^	4.43 ± 1.00
TG, mean ± SD, mmol/L	1.54 ± 0.79	1.64 ± 1.05	1.74 ± 1.21
LDL-C, mean ± SD, mmol/L	2.79 ± 0.70	2.46 ± 0.83^∗∗^	2.73 ± 0.85
HDL, mean ± SD, mmol/L	1.06 ± 0.20	1.02 ± 0.23	1.04 ± 0.20
Admission NIHSS, median (IQR)	5 (4-8)	5 (4-7)	6 (4.25-7)
mRS at 3 months, median (IQR)	2 (1-4)	2 (1-4)	2 (1-4)
Infarct volume, mL, median (IQR)	2.02 (1.50-4.64)	2.16 (1.43-5.58)	1.95 (1.50-3.97)
Stroke subtype (TOAST)			
A large vessel, *n* (%)	35 (32.7)	12 (17.4)	14 (25.0)
A small vessel, *n* (%)	45 (42.1)	25 (36.2)	21 (37.5)
Cardioembolic, *n* (%)	15 (14.0)	24 (34.8)	15 (26.8)
Other reasons, *n* (%)	2 (1.9)	1 (1.4)	1 (1.8)
Undetermined, *n* (%)	10 (9.3)	7 (10.1)	5 (8.9)
Leukocytes (×10^9^)	7.17 ± 1.95	7.74 ± 2.25	7.72 ± 2.08
Neutrophils (×10^9^)	4.79 ± 1.76	5.15 ± 2.23	5.28 ± 1.77
Lymphocytes (×10^9^)	1.70 ± 0.53	1.92 ± 0.69^∗^	1.70 ± 0.68
Monocytes (×10^9^)	0.52 ± 0.17	0.49 ± 0.16	0.53 ± 0.19
Eosinophils (×10^9^)	0.15 ± 0.12	0.16 ± 0.13	0.16 ± 0.13
Basophils (×10^9^)	0.02 ± 0.02	0.02 ± 0.02	0.03 ± 0.03
NLR	3.18 ± 1.79	3.27 ± 2.58	3.48 ± 1.74
LMR	3.48 ± 1.18	4.17 ± 1.78^∗∗^	3.55 ± 1.63

^∗^ *p* < 0.05, matched controls versus beta-blockers. ^∗∗^*p* < 0.01, matched controls versus beta-blockers. ^∗∗∗^*p* < 0.001, matched controls versus beta-blockers. ^†^*p* < 0.05, matched controls versus ACEI/ARB. ^††^*p* < 0.01, matched controls versus ACEI/ARB. ^†††^*p* < 0.001, matched controls versus ACEI/ARB. SD: standard deviation; IQR: interquartile range; NIHSS: National Institutes of Health Stroke Scale score; TC: total cholesterol; TG: triglyceride; LDL-C: low-density lipoprotein cholesterol; HDL: high-density lipoprotein cholesterol; NLR: neutrophil-to-lymphocyte ratio; LMR: lymphocyte-to-monocyte ratio.

**Table 2:** Univariate analysis for the characteristics of patients with premorbid use of beta-blockers and matched controls.

Characteristic and variables	mRS ≤ 3(*n* = 125)	mRS > 3 (*n* = 51)
Male, gender, *n* (%)	81 (64.8)	30 (58.8)
Age, (years, mean ± SD)	60.9 ± 12.0	67.2 ± 12.4^∗∗^
Hypertension, *n* (%)	92 (73.6)	36 (70.6)
Coronary heart disease, *n* (%)	33 (26.4)	26 (51.0)^∗∗^
Atrial fibrillation, *n* (%)	27 (21.6)	22 (43.1)^∗∗^
Diabetes, *n* (%)	15 (12.0)	16 (31.4)^∗∗^
Hyperlipidemia, *n* (%)	55 (44.0)	17 (33.3)
Previous stroke, *n* (%)	24 (19.2)	11 (21.6)
Prior antiplatelets, *n* (%)	31 (24.8)	9 (17.6)
Prior statins, *n* (%)	26 (20.8)	9 (17.6)
Smoking, *n* (%)	57 (45.6)	17 (33.3)
Alcoholic drinking, *n* (%)	47 (37.6)	13 (25.5)
Infection while hospitalized, *n* (%)	19 (15.2)	25 (49.0)^∗∗∗^
Prior beta-blockers, *n* (%)	49 (39.2)	20 (39.2)
Systolic blood pressure at admission, mean ± SD, mmHg	152.0 ± 23.5	156.8 ± 22.2
Diastolic blood pressure at admission, mean ± SD, mmHg	89.5 ± 15.7	90.4 ± 14.5
Fasting blood glucose, mean ± SD, mmol/L	5.91 ± 2.02	6.19 ± 1.94
Admission blood lipid		
TC, mean ± SD, mmol/L	4.37 ± 0.85	4.27 ± 1.04
TG, mean ± SD, mmol/L	1.59 ± 0.89	1.55 ± 0.94
LDL-C, mean ± SD, mmol/L	2.67 ± 0.76	2.64 ± 0.81
HDL, mean ± SD, mmol/L	1.05 ± 0.21	1.03 ± 0.23
Admission NIHSS, median (IQR)	4 (3-6)	9 (7-13)^∗∗∗^
Infarct volume, mL, median (IQR)	1.72 (1.32-2.53)	7.24 (2.28-28.92)^∗∗∗^
Stroke subtype (TOAST)		
Large vessel, *n* (%)	28 (22.4)	19 (37.3)
Small vessel, *n* (%)	61 (48.8)	9 (17.6)
Cardioembolic, *n* (%)	23 (18.4)	16 (31.4)
Other reasons, *n* (%)	2 (1.6)	1 (2.0)
Undetermined, *n* (%)	11 (8.8)	6 (11.8)
Leukocytes (×10^9^)	6.94 ± 1.79	8.52 ± 2.32^∗∗∗^
Neutrophils (×10^9^)	4.43 ± 1.67	6.15 ± 2.09^∗∗∗^
Lymphocytes (×10^9^)	1.81 ± 0.53	1.72 ± 0.75
Monocytes (×10^9^)	0.52 ± 0.14	0.49 ± 0.21
Eosinophils (×10^9^)	0.16 ± 0.12	0.14 ± 0.13
Basophils (×10^9^)	0.02 ± 0.02	0.02 ± 0.02
NLR	2.75 ± 1.73	4.34 ± 2.56^∗∗∗^
LMR	3.69 ± 1.24	3.91 ± 1.95

^∗∗^ *p* < 0.01, mRS ≤ 3 versus mRS > 3; ^∗∗∗^*p* < 0.001, mRS ≤ 3 versus mRS > 3. SD: standard deviation; IQR: interquartile range; NIHSS: National Institutes of Health Stroke Scale score; TC: total cholesterol; TG: triglyceride; LDL-C: low-density lipoprotein cholesterol; HDL: high-density lipoprotein cholesterol; NLR: neutrophil-to-lymphocyte ratio; LMR: lymphocyte-to-monocyte ratio.

**Table 3:** Univariate analysis for the characteristics of patients with premorbid use of ACEIs/ARBs and matched controls.

Characteristic and variables	mRS ≤ 3 (*n* = 117)	mRS > 3 (*n* = 46)
Male, gender, *n* (%)	77 (65.8)	31 (67.4)
Age, (years, mean ± SD)	60.3 ± 10.9	66.1 ± 12.7^∗∗^
Hypertension, *n* (%)	87 (74.4)	33 (71.7)
Coronary heart disease, *n* (%)	24 (20.5)	16 (34.8)
Atrial fibrillation, *n* (%)	20 (17.1)	17 (37.0)^∗∗^
Diabetes, *n* (%)	17 (14.5)	13 (28.3)^∗^
Hyperlipidemia, *n* (%)	54 (46.2)	18 (39.1)
Previous stroke, *n* (%)	22 (18.8)	13 (28.3)
Prior antiplatelets, *n* (%)	15 (12.8)	7 (15.2)
Prior statins, *n* (%)	12 (10.3)	7 (15.2)
Smoking, *n* (%)	55 (47.0)	18 (39.1)
Alcoholic drinking, *n* (%)	43 (36.8)	15 (32.6)
Infection while hospitalized, *n* (%)	23 (19.7)	23 (50.0)^∗∗∗^
Prior ACEI/ARB, *n* (%)	41 (35.0)	15 (32.6)
Systolic blood pressure at admission, mean ± SD, mmHg	153.1 ± 25.8	157.6 ± 21.0
Diastolic blood pressure at admission, mean ± SD, mmHg	89.4 ± 14.8	91.7 ± 13.2
Fasting blood glucose, mean ± SD, mmol/L	5.82 ± 2.06	6.30 ± 2.00
Blood lipid on admission		
TC, mean ± SD, mmol/L	4.48 ± 0.79	4.44 ± 1.11
TG, mean ± SD, mmol/L	1.60 ± 0.82	1.64 ± 1.26
LDL-C, mean ± SD, mmol/L	2.78 ± 0.69	2.76 ± 0.90
HDL, mean ± SD, mmol/L	1.05 ± 0.20	1.05 ± 0.21
NIHSS on admission, median (IQR)	5 (4-6)	9 (6.75-12)^∗∗∗^
Infarct volume, mL, median (IQR)	1.72 (1.36-2.72)	6.08 (2.24-17.50)^∗∗∗^
Stroke subtype (TOAST)		
Large vessel, *n* (%)	32 (27.4)	17 (37.0)
Small vessel, *n* (%)	57 (48.7)	9 (19.6)
Cardioembolic, *n* (%)	18 (15.4)	12 (26.1)
Other reasons, *n* (%)	1 (0.9)	2 (4.3)
Undetermined, *n* (%)	9 (7.7)	6 (13.0)
Leukocytes (×10^9^)	7.16 ± 1.86	7.87 ± 2.27^∗^
Neutrophils (×10^9^)	4.73 ± 1.67	5.53 ± 1.92^∗^
Lymphocytes (×10^9^)	1.74 ± 0.55	1.59 ± 0.65
Monocytes (×10^9^)	0.51 ± 0.16	0.55 ± 0.22
Eosinophils (×10^9^)	0.15 ± 0.12	0.16 ± 0.15
Basophils (×10^9^)	0.02 ± 0.02	0.02 ± 0.02
NLR	3.00 ± 1.53	4.00 ± 2.13^∗∗^
LMR	3.66 ± 1.38	3.10 ± 1.18^∗^

^∗^ *p* < 0.05, mRS ≤ 3 versus mRS > 3. ^∗∗^*p* < 0.01, mRS ≤ 3 versus mRS > 3. ^∗∗∗^*p* < 0.001, mRS ≤ 3 versus mRS > 3. SD: standard deviation; IQR: interquartile range; NIHSS: National Institutes of Health Stroke Scale score; TC: total cholesterol; TG: triglyceride; LDL-C: low-density lipoprotein cholesterol; HDL: high-density lipoprotein cholesterol; NLR: neutrophil-to-lymphocyte ratio; LMR: lymphocyte-to-monocyte ratio.

## Data Availability

All relevant data from this study are included in the article and its supplementary materials.

## Supplementary Materials

Supplementary Materials Supplementary Table 1: baseline data for healthy controls and matched controls. Supplementary Table 2: dosages of different beta-blockers and ACEIs/ARBs used prior to stroke onset expressed as percentages of maximum recommended therapeutic dose. Supplementary File 1: raw data from patients in the healthy control group, the matched control group, the beta-blocker group, and the ACEI/ARB group.

## References

[1] Campbell B. C. V., Khatri P. (2020). Stroke. *Lancet*.

[2] Jiang C., Zuo F., Wang Y., Lu H., Yang Q., Wang J. (2017). Progesterone changes VEGF and BDNF expression and promotes neurogenesis after ischemic stroke. *Molecular Neurobiology*.

[3] Jiang C., Wang J., Yu L. (2013). Comparison of the therapeutic effects of bone marrow mononuclear cells and microglia for permanent cerebral ischemia. *Behavioural Brain Research*.

[4] Powers W. J., Rabinstein A. A., Ackerson T. (2019). Guidelines for the early management of patients with acute ischemic stroke: 2019 update to the 2018 guidelines for the early management of acute ischemic stroke: a guideline for healthcare professionals from the American Heart Association/American Stroke Association. *Stroke*.

[5] Gan Y., Wu J., Zhang S. (2017). Prevalence and risk factors associated with stroke in middle-aged and older Chinese: a community-based cross-sectional study. *Scientific Reports*.

[6] Moskowitz M. A., Lo E. H., Iadecola C. (2010). The science of stroke: mechanisms in search of treatments. *Neuron*.

[7] Petrovic-Djergovic D., Goonewardena S. N., Pinsky D. J. (2016). Inflammatory disequilibrium in stroke. *Circulation Research*.

[8] Macrez R., Ali C., Toutirais O. (2011). Stroke and the immune system: from pathophysiology to new therapeutic strategies. *Lancet Neurology*.

[9] Kim J. Y., Kawabori M., Yenari M. A. (2014). Innate inflammatory responses in stroke: mechanisms and potential therapeutic targets. *Current Medicinal Chemistry*.

[10] Zhu L., Huang L., Le A. (2022). Interactions between the autonomic nervous system and the immune system after stroke. *Comprehensive Physiology*.

[11] Urra X., Laredo C., Zhao Y. (2017). Neuroanatomical correlates of stroke-associated infection and stroke-induced immunodepression. *Brain, Behavior, and Immunity*.

[12] Yan F. L., Zhang J. H. (2014). Role of the sympathetic nervous system and spleen in experimental stroke-induced immunodepression. *Medical Science Monitor*.

[13] Doyle K. P., Quach L. N., Sole M. (2015). B-lymphocyte-mediated delayed cognitive impairment following stroke. *The Journal of Neuroscience*.

[14] Selvaraj U. M., Poinsatte K., Torres V., Ortega S. B., Stowe A. M. (2016). Heterogeneity of B cell functions in stroke-related risk, prevention, injury, and repair. *Neurotherapeutics*.

[15] Westendorp W. F., Dames C., Nederkoorn P. J., Meisel A. (2022). Immunodepression, infections, and functional outcome in ischemic stroke. *Stroke*.

[16] Courties G., Herisson F., Sager H. B. (2015). Ischemic stroke activates hematopoietic bone marrow stem cells. *Circulation Research*.

[17] Liberale L., Montecucco F., Bonaventura A. (2017). Monocyte count at onset predicts poststroke outcomes during a 90-day follow-up. *European Journal of Clinical Investigation*.

[18] Pagram H., Bivard A., Lincz L. F., Levi C. (2016). Peripheral immune cell counts and advanced imaging as biomarkers of stroke outcome. *Cerebrovascular Diseases Extra*.

[19] Qun S., Tang Y., Sun J. (2017). Neutrophil-to-lymphocyte ratio predicts 3-month outcome of acute ischemic stroke. *Neurotoxicity Research*.

[20] Ren H., Liu X., Wang L., Gao Y. (2017). Lymphocyte-to-monocyte ratio: a novel predictor of the prognosis of acute ischemic stroke. *Journal of Stroke and Cerebrovascular Diseases*.

[21] Wang H., Deng Q. W., Peng A. N. (2018). *β*-arrestin2 functions as a key regulator in the sympathetic-triggered immunodepression after stroke. *Journal of Neuroinflammation*.

[22] Martin N., Manoharan K., Thomas J., Davies C., Lumbers R. T., Cochrane Heart Group (2018). Beta-blockers and inhibitors of the renin-angiotensin aldosterone system for chronic heart failure with preserved ejection fraction. *Cochrane Database of Systematic Reviews*.

[23] Swirski F. K., Nahrendorf M., Etzrodt M. (2009). Identification of splenic reservoir monocytes and their deployment to inflammatory sites. *Science*.

[24] Heart Outcomes Prevention Evaluation Study Investigators, Yusuf S., Sleight P. (2000). Effects of an angiotensin-converting-enzyme inhibitor, ramipril, on cardiovascular events in high-risk patients. *The New England Journal of Medicine*.

[25] Sylaja P. N., Nair S. S., Pandian J. (2021). Impact of pre-stroke antiplatelet use on 3-month outcome after ischemic stroke. *Neurology India*.

[26] Viktorisson A., Reinholdsson M., Danielsson A., Palstam A., Sunnerhagen K. S. (2022). Pre-stroke physical activity in relation to post-stroke outcomes - linked to the international classification of functioning, disability and health (ICF): a scoping review. *Journal of Rehabilitation Medicine*.

[27] Wankowicz P., Staszewski J., Debiec A. (2021). Pre-stroke statin therapy improves in-hospital prognosis following acute ischemic stroke associated with well-controlled nonvalvular atrial fibrillation. *Journal of Clinical Medicine*.

[28] Kersten C., Knottnerus I. L. H., Heijmans E., Haalboom M., Zandbergen A. A. M., den Hertog H. M. (2022). Effect of metformin on outcome after acute ischemic stroke in patients with type 2 diabetes mellitus. *Journal of Stroke and Cerebrovascular Diseases*.

[29] Adams H. P., Bendixen B. H., Kappelle L. J. (1993). Classification of subtype of acute ischemic stroke. Definitions for use in a multicenter clinical trial. TOAST. Trial of org 10172 in acute stroke treatment. *Stroke*.

[30] Sims J. R., Gharai L. R., Schaefer P. W. (2009). ABC/2 for rapid clinical estimate of infarct, perfusion, and mismatch volumes. *Neurology*.

[31] Brott T., Marler J. R., Olinger C. P. (1989). Measurements of acute cerebral infarction: lesion size by computed tomography. *Stroke*.

[32] Bonita R., Beaglehole R. (1988). Recovery of motor function after stroke. *Stroke*.

[33] Venema E., Mulder M., Roozenbeek B. (2017). Selection of patients for intra-arterial treatment for acute ischaemic stroke: development and validation of a clinical decision tool in two randomised trials. *BMJ*.

[34] Wilson D., Charidimou A., Shakeshaft C., Ambler G., Werring D. J. (2015). Volume and functional outcome of intracerebral hemorrhage according to oral anticoagulant type. *Neurology*.

[35] O'Connell G. C., Chang J. H. C. (2018). Analysis of early stroke-induced changes in circulating leukocyte counts using transcriptomic deconvolution. *Translational Neuroscience*.

[36] Anrather J., Iadecola C. (2016). Inflammation and stroke: an overview. *Neurotherapeutics*.

[37] Hooman K., Costantino I. (2012). Brain-immune interactions and ischemic stroke. *Archives of Neurology*.

[38] Liesz A., Hu X., Kleinschnitz C., Offner H. (2015). Functional role of regulatory lymphocytes in stroke: facts and controversies. *Stroke*.

[39] Zhu H., Wang Z., Yu J. (2019). Role and mechanisms of cytokines in the secondary brain injury after intracerebral hemorrhage. *Progress in Neurobiology*.

[40] Urra X., Cervera A., Obach V., Climent N., Planas A. M., Chamorro A. (2009). Monocytes are major players in the prognosis and risk of infection after acute stroke. *Stroke*.

[41] Miro-Mur F., Laredo C., Renu A. (2018). Adrenal hormones and circulating leukocyte subtypes in stroke patients treated with reperfusion therapy. *Brain, Behavior, and Immunity*.

[42] Mracsko E., Liesz A., Karcher S., Zorn M., Bari F., Veltkamp R. (2014). Differential effects of sympathetic nervous system and hypothalamic-pituitary- adrenal axis on systemic immune cells after severe experimental stroke. *Brain, Behavior, and Immunity*.

[43] Sander D., Winbeck K., Klingelhofer J., Etgen T., Conrad B. (2001). Prognostic relevance of pathological sympathetic activation after acute thromboembolic stroke. *Neurology*.

[44] Kangussu L. M., Marzano L. A. S., Souza C. F., Dantas C. C., Miranda A. S., Simoes E. S. A. C. (2020). The renin-angiotensin system and the cerebrovascular diseases: experimental and clinical evidence. *Protein and Peptide Letters*.

[45] Elenkov I. J., Wilder R. L., Chrousos G. P., Vizi E. S. (2000). The sympathetic nerve--an integrative interface between two supersystems: the brain and the immune system. *Pharmacological Reviews*.

[46] Benigni A., Cassis P., Remuzzi G. (2010). Angiotensin II revisited: new roles in inflammation, immunology and aging. *EMBO Molecular Medicine*.

[47] Kohm A. P., Sanders V. M. (2001). Norepinephrine and beta 2^–^ adrenergic receptor stimulation regulate CD4^+^ T and B lymphocyte function in vitro and in vivo. *Pharmacological Reviews*.

[48] Marra S., Hoffman-Goetz L. (2004). Beta-adrenergic receptor blockade during exercise decreases intestinal lymphocyte apoptosis but not cell loss in mice. *Canadian Journal of Physiology and Pharmacology*.

[49] Prass K., Meisel C., Höflich C. (2003). Stroke-induced immunodeficiency promotes spontaneous bacterial infections and is mediated by sympathetic activation reversal by poststroke T helper cell type 1-like immunostimulation. *The Journal of Experimental Medicine*.

[50] Shim R., Wong C. H. Y. (2018). Complex interplay of multiple biological systems that contribute to post- stroke infections. *Brain, Behavior, and Immunity*.

[51] Jiang C., Wang Y., Hu Q. (2020). Immune changes in peripheral blood and hematoma of patients with intracerebral hemorrhage. *The FASEB Journal*.

[52] Ito M., Komai K., Mise-Omata S. (2019). Brain regulatory T cells suppress astrogliosis and potentiate neurological recovery. *Nature*.

[53] Kanazawa M., Ninomiya I., Hatakeyama M., Takahashi T., Shimohata T. (2017). Microglia and monocytes/macrophages polarization reveal novel therapeutic mechanism against stroke. *International Journal of Molecular Sciences*.

[54] Grailer J. J., Haggadone M. D., Sarma J. V., Zetoune F. S., Ward P. A. (2014). induction of M2 regulatory macrophages through the *β*_2_-adrenergic receptor with protection during endotoxemia and acute lung injury. *Journal of Innate Immunity*.

[55] Courties G., Frodermann V., Honold L. (2019). Glucocorticoids regulate bone marrow B lymphopoiesis after stroke. *Circulation Research*.

[56] Olsson T. (1990). Urinary free cortisol excretion shortly after ischaemic stroke. *Journal of Internal Medicine*.

[57] Donadelli R., Abbate M., Zanchi C. (2000). Protein traffic activates NF-kB gene signaling and promotes MCP-1-dependent interstitial inflammation. *American Journal of Kidney Diseases*.

[58] Gao X. M., Tsai A., Al-Sharea A. (2017). Inhibition of the renin-angiotensin system post myocardial infarction prevents inflammation-associated acute cardiac rupture. *Cardiovascular Drugs and Therapy*.

[59] Bryniarski P., Nazimek K., Marcinkiewicz J. (2022). Immunomodulatory activity of the most commonly used antihypertensive drugs-angiotensin converting enzyme inhibitors and angiotensin II receptor blockers. *International Journal of Molecular Sciences*.

[60] Panico K., Abrahao M. V., Trentin-Sonoda M., Muzi-Filho H., Vieyra A., Carneiro-Ramos M. S. (2019). Cardiac inflammation after ischemia-reperfusion of the kidney: role of the sympathetic nervous system and the renin-angiotensin system. *Cellular Physiology and Biochemistry*.

[61] Saavedra J. M., Julius B. (2007). Brain and peripheral angiotensin II play a major role in stress. *Stress-the International Journal on the Biology of Stress*.

[62] Sánchez-Lemus E., Benicky J., Pavel J., Saavedra J. M. (2009). *In vivo* angiotensin II AT_1_ receptor blockade selectively inhibits LPS-induced innate immune response and ACTH release in rat pituitary gland. *Brain, Behavior, and Immunity*.

[63] Prass K., Braun J. S., Dirnagl U., Meisel C., Meisel A. (2006). Stroke propagates bacterial aspiration to pneumonia in a model of cerebral ischemia. *Stroke*.

[64] Kalita J., Misra U. K., Kumar B. (2013). Is beta-blocker (atenolol) a preferred antihypertensive in acute intracerebral hemorrhage?. *Neurological Sciences*.

[65] Phelan C., Alaigh V., Fortunato G., Staff I., Sansing L. (2015). Effect of *β*-adrenergic antagonists on in-hospital mortality after ischemic stroke. *Journal of Stroke and Cerebrovascular Diseases*.

[66] Maier I. L., Becker J. C., Leyhe J. R. (2018). Influence of beta-blocker therapy on the risk of infections and death in patients at high risk for stroke induced immunodepression. *PLoS One*.

[67] De Raedt S., Haentjens P., De Smedt A. (2012). Pre-stroke use of beta-blockers does not affect ischaemic stroke severity and outcome. *European Journal of Neurology*.

[68] Koton S., Tanne D., Grossman E. (2017). Prestroke treatment with beta-blockers for hypertension is not associated with severity and poor outcome in patients with ischemic stroke. *Journal of Hypertension*.

[69] Jesse W. (2007). Morbidity and mortality after stroke--eposartan compared with nitrendipine for secondary prevention: principal results of a prospective randomized controlled study (MOSES). *Current Cardiology Reports*.

[70] Czernichow S., Ninomiya T., Huxley R. (2010). Impact of blood pressure lowering on cardiovascular outcomes in normal weight, overweight, and obese Individuals. *Hypertension*.

[71] Nagai M., Terao S., Vital S. A., Rodrigues S. F., Yilmaz G., Granger D. N. (2011). Role of blood cell-associated angiotensin II type 1 receptors in the cerebral microvascular response to ischemic stroke during angiotensin-induced hypertension. *Experimental & Translational Stroke Medicine*.

[72] Elizabeth H. W., Emma T., Robert V. (2010). Angiotensin-converting enzyme (ACE) inhibitors exacerbate histological damage and motor deficits after experimental traumatic brain injury. *Neuroscience Letters*.

