# Supplementary material for: Premorbid Use of Beta-Blockers or Angiotensin-Converting Enzyme Inhibitors/Angiotensin Receptor Blockers in Patients with Acute Ischemic Stroke
Source: Oxid Med Cell Longev. 2023 Feb 1;2023:7733857. doi: 10.1155/2023/7733857 (PMC9908343; doi:10.1155/2023/7733857)
Supplement: Supplementary Materials — Supplementary Table 1: baseline data for healthy controls and matched controls. Supplementary Table 2: dosages of different beta-blockers and ACEIs/ARBs used prior to stroke onset expressed as percentages of maximum recommended therapeutic dose. Supplementary File 1: raw data from patients in the healthy control group, the matched control group, the beta-blocker group, and the ACEI/ARB group. [file 7733857.f1.zip › Supplementary file 1.pdf]

1).Raw data for patients in healthy control group

Patient No.	Drug	Dosage	Leukocytes(x109)	Neutroplils(x109)
1	NA	NA	7.95	5.7
2	NA	NA	7.85	4.7
3	NA	NA	13.35	11.6
4	NA	NA	10.35	8.6
5	NA	NA	5.65	3.5
6	NA	NA	9.45	5.9
7	NA	NA	6.15	3.7
8	NA	NA	4.75	2.4
9	NA	NA	6.25	3.3
10	NA	NA	6.55	4.2
11	NA	NA	5.05	3
12	NA	NA	6.25	3.4
13	NA	NA	6.35	4
14	NA	NA	7.95	4.3
15	NA	NA	5.95	3.5
16	NA	NA	6.45	3.4
17	NA	NA	6.15	3.3
18	NA	NA	6.85	4.4
19	NA	NA	6.05	3.1
20	NA	NA	6.49	4.47
21	NA	NA	6.15	3.9
22	NA	NA	4.95	3.1
23	NA	NA	5.15	2.9
24	NA	NA	7.05	4.6
25	NA	NA	5.15	3.1
26	NA	NA	7.45	4.2
27	NA	NA	8.65	4.8
28	NA	NA	5.85	2.9
29	NA	NA	4.75	3.07
30	NA	NA	5.54	3.04
31	NA	NA	7.26	4.02
32	NA	NA	4.48	1.84
33	NA	NA	6.85	3.69
34	NA	NA	6.16	4.12
35	NA	NA	9.79	6.26
36	NA	NA	4.62	2.83
37	NA	NA	4.5	2.94
38	NA	NA	5.94	4.03
39	NA	NA	7.49	3.82
40	NA	NA	7.08	4
41	NA	NA	5.89	3.28
42	NA	NA	5.62	3.81
43	NA	NA	6.76	3.43
44	NA	NA	4.9	3.04
45	NA	NA	8.08	5.05

Lymphocytes(x109)	Monocytes(x109)	Eosinophils(x109)	Basophils(x109)	NLR
1.55	0.6	0.1	0	3.677419
2.55	0.5	0.1	0	1.843137
1.25	0.5	0.1	0.02	9.28
1.35	0.4	0	0	6.37037
1.65	0.3	0.1	0	2.121212
2.55	0.7	0.2	0	2.313725
2.05	0.3	0.1	0	1.804878
1.75	0.4	0.1	0	1.371429
2.15	0.6	0.1	0	1.534884
1.85	0.4	0	0	2.27027
1.65	0.3	0.1	0	1.818182
2.15	0.5	0.1	0	1.581395
1.65	0.4	0.3	0	2.424242
2.95	0.5	0.2	0.1	1.457627
1.95	0.3	0.2	0	1.794872
2.65	0.3	0.1	0.1	1.283019
2.15	0.5	0.2	0	1.534884
1.65	0.4	0.4	0	2.666667
2.45	0.5	0.1	0	1.265306
1.63	0.3	0.07	0.02	2.742331
1.65	0.3	0.2	0	2.363636
1.35	0.3	0.1	0	2.296296
1.55	0.6	0.1	0	1.870968
2.15	0.3	0	0	2.139535
1.65	0.3	0	0	1.878788
2.75	0.5	0.1	0	1.527273
3.05	0.5	0.2	0	1.57377
2.25	0.4	0.3	0	1.288889
1.3	0.16	0.21	0.01	2.361538
0.38	1.99	0.09	0.03	8
0.45	2.67	0.09	0.04	8.933333
2.14	0.3	0.18	0.03	0.859813
2.51	0.59	0.04	0.03	1.47012
1.49	0.46	0.05	0.03	2.765101
2.96	0.51	0.03	0.03	2.114865
1.29	0.28	0.18	0.03	2.193798
1.16	0.32	0.06	0.02	2.534483
1.22	0.48	0.2	0.02	3.303279
3.05	0.46	0.13	0.03	1.252459
2.17	0.61	0.27	0.03	1.843318
1.93	0.52	0.13	0.04	1.699482
1.42	0.27	0.12	0	2.683099
2.58	0.49	0.21	0.03	1.329457
1.33	0.43	0.09	0.01	2.285714
2.42	0.39	0.15	0.07	2.086777

LMR	NIHSS	mRS	Infarct volume	Gender	Age	Hypertension
2.583333	NA	NA	NA	Female	54	No
5.1	NA	NA	NA	Male	48	Yes
2.5	NA	NA	NA	Male	46	No
3.375	NA	NA	NA	Female	49	No
5.5	NA	NA	NA	Female	59	No
3.642857	NA	NA	NA	Male	43	No
6.833333	NA	NA	NA	Male	46	No
4.375	NA	NA	NA	Male	61	No
3.583333	NA	NA	NA	Male	60	No
4.625	NA	NA	NA	Male	52	No
5.5	NA	NA	NA	Female	53	No
4.3	NA	NA	NA	Male	62	No
4.125	NA	NA	NA	Female	57	No
5.9	NA	NA	NA	Female	67	No
6.5	NA	NA	NA	Female	68	Yes
8.833333	NA	NA	NA	Female	60	No
4.3	NA	NA	NA	Female	44	No
4.125	NA	NA	NA	Female	43	No
4.9	NA	NA	NA	Male	61	No
5.433333	NA	NA	NA	Male	53	No
5.5	NA	NA	NA	Female	53	No
4.5	NA	NA	NA	Female	56	No
2.583333	NA	NA	NA	Male	49	No
7.166667	NA	NA	NA	Female	53	No
5.5	NA	NA	NA	Male	63	Yes
5.5	NA	NA	NA	Female	50	No
6.1	NA	NA	NA	Male	64	No
5.625	NA	NA	NA	Male	69	No
8.125	NA	NA	NA	Male	43	No
0.190955	NA	NA	NA	Female	50	No
0.168539	NA	NA	NA	Male	43	No
7.133333	NA	NA	NA	Female	84	No
4.254237	NA	NA	NA	Female	72	No
3.23913	NA	NA	NA	Male	80	Yes
5.803922	NA	NA	NA	Male	69	Yes
4.607143	NA	NA	NA	Male	81	Yes
3.625	NA	NA	NA	Male	57	No
2.541667	NA	NA	NA	Male	78	No
6.630435	NA	NA	NA	Male	68	No
3.557377	NA	NA	NA	Male	69	Yes
3.711538	NA	NA	NA	Male	65	Yes
5.259259	NA	NA	NA	Female	69	Yes
5.265306	NA	NA	NA	Female	70	No
3.093023	NA	NA	NA	Female	84	No
6.205128	NA	NA	NA	Female	87	Yes

Admission SP	Admission DP	Coronary heart disease	Atrial fibrillation	Diabetes
120	70	No	No	No
140	85	No	No	No
116	70	No	No	No
135	86	No	No	No
120	70	No	No	No
128	80	No	No	No
120	75	No	No	No
140	90	No	No	No
135	78	No	No	No
150	90	No	Yes	No
118	62	No	No	No
158	98	No	No	No
185	95	Yes	No	No
145	80	No	No	Yes
120	60	No	No	No
110	70	No	No	No
128	78	No	No	No
108	80	No	No	No
136	86	No	No	No
120	80	No	No	No
130	80	No	No	No
100	60	No	No	No
142	90	No	No	No
150	90	No	No	No
130	80	No	No	No
110	60	No	No	No
120	75	No	No	No
150	80	No	No	No
130	70	No	No	No
120	80	No	No	No
128	92	No	No	No
144	86	No	No	Yes
156	87	No	No	No
124	72	Yes	No	No
175	110	Yes	No	No
142	80	No	No	No
130	70	No	No	No
170	90	No	No	No
110	60	Yes	No	No
130	80	Yes	No	Yes
130	75	Yes	No	No
190	90	No	No	No
150	90	No	No	No
130	70	No	No	No
170	75	No	No	No

Hyperlipidemia	Previous stroke	Fasting blood glucose	TC	TG	LDL
No	No	6.4	5.1	0.55	3.05
No	No	5.71	4.63	1.64	3.26
Yes	No	3.56	4.87	1.42	3.66
Yes	No	4.91	4	2.12	2.31
Yes	No	5.59	5.45	1.21	3.71
No	No	4.62	4.97	0.85	2.68
Yes	No	4.51	7.15	2.82	4.41
No	No	4.66	4.66	1.46	2.87
No	No	5.5	2.69	1.05	1.6
No	No	5.23	4.04	1.1	2.49
No	No	5.17	4.44	1.56	2.55
Yes	No	4.21	5.19	1.25	3.59
No	No	4.73	4.12	1.19	2.61
No	No	5.25	3.88	0.94	1.97
No	No	4.75	4.37	0.95	2.19
No	No	5.61	4.42	0.7	2.95
No	No	4.21	4.39	0.85	2.73
No	No	4.59	3.99	1.06	2.46
Yes	No	4.06	5.22	1.19	3.51
Yes	No	5.64	7.17	2.01	4.67
Yes	No	3.57	5.71	1.21	3.87
Yes	No	5.21	5.45	1.07	3.65
No	No	5.3	4.35	0.69	2.7
No	No	4.84	4.24	0.89	2.67
No	No	4.85	3.8	1.69	1.92
Yes	No	4.56	5.34	1.12	3.28
Yes	No	5.41	3.63	2.18	1.95
No	No	4.88	4.7	0.87	3
No	No	4.57	4.61	1.55	3.14
No	No	4.78	4.51	0.57	2.67
Yes	No	14.37	4.72	6.28	2.24
Yes	Yes	4.4	5.65	1.16	3.13
No	No	4.75	5.11	0.88	3.31
No	Yes	3.77	2.72	0.98	1.35
No	No	4.72	4.33	1.45	2.69
No	No	4.85	3.66	1.42	1.95
No	No	4.02	4.96	1.66	3.06
No	Yes	4.17	3.04	0.66	1.49
No	No	4.85	5.11	1.17	2.93
Yes	No	10.03	4.07	1.97	1.81
No	No	5.34	5.12	0.8	3.13
No	No	4.53	4.19	1	2.67
No	No	5.97	4.98	1.41	2.92
Yes	No	4.95	4.88	1.76	2.85
Yes	No	4.99	8.05	2.57	4.97

HDL	Smoking	Alcohol drinking	Prior anti-platelets	Prior statins
1.7	No	No	No	No
0.95	Yes	Yes	No	No
1.13	Yes	Yes	No	No
1.2	No	No	No	No
1.41	No	No	No	No
1.59	Yes	Yes	No	No
1.21	No	Yes	No	No
0.88	No	No	No	No
0.69	Yes	No	No	No
0.94	No	No	No	No
1.3	No	No	No	No
0.91	No	No	No	No
1.15	No	No	No	No
1.32	No	No	No	No
1.5	No	No	No	No
1.16	No	No	No	No
1.04	No	No	No	No
1.07	No	No	No	No
1.19	No	No	No	No
1.28	Yes	Yes	No	No
1.27	No	No	No	No
1.33	No	No	No	No
1.13	No	No	No	No
1.14	No	No	No	No
1.23	No	No	No	No
1.48	No	No	No	No
0.98	Yes	No	No	No
1.1	No	No	No	No
0.81	Yes	No	No	No
1.41	No	No	No	No
0.79	No	No	No	No
1.55	No	No	No	No
1.38	No	No	No	No
0.91	No	No	No	No
0.81	No	No	No	No
1.02	No	No	No	No
0.99	No	No	No	No
1.16	No	No	Yes	Yes
1.21	No	Yes	No	No
1.08	Yes	Yes	No	No
1.22	No	No	No	No
1	No	No	No	No
1.11	No	No	No	No
0.83	No	No	No	No
1.49	No	No	No	No

Infection while in hospital	ACS/PCS	TOAST
NA	NA	NA
NA	NA	NA
NA	NA	NA
NA	NA	NA
NA	NA	NA
NA	NA	NA
NA	NA	NA
NA	NA	NA
NA	NA	NA
NA	NA	NA
NA	NA	NA
NA	NA	NA
NA	NA	NA
NA	NA	NA
NA	NA	NA
NA	NA	NA
NA	NA	NA
NA	NA	NA
NA	NA	NA
NA	NA	NA
NA	NA	NA
NA	NA	NA
NA	NA	NA
NA	NA	NA
NA	NA	NA
NA	NA	NA
NA	NA	NA
NA	NA	NA
NA	NA	NA
NA	NA	NA
NA	NA	NA
NA	NA	NA
NA	NA	NA
NA	NA	NA
NA	NA	NA
NA	NA	NA
NA	NA	NA
NA	NA	NA
NA	NA	NA
NA	NA	NA
NA	NA	NA
NA	NA	NA
NA	NA	NA
NA	NA	NA
NA	NA	NA

2).Raw data for patients in matched control group

Patient No.	Drug	Dosage	Leukocytes(x109)	Neutroplils(x109)
1	NA	NA	5.54	3.25
2	NA	NA	6.82	4.93
3	NA	NA	7.35	4.95
4	NA	NA	7.16	4.63
5	NA	NA	5.76	2.93
6	NA	NA	5.14	3.74
7	NA	NA	5.76	3.63
8	NA	NA	4.74	3.4
9	NA	NA	5.31	2.59
10	NA	NA	8.86	5.33
11	NA	NA	5.96	3.53
12	NA	NA	11.36	9.13
13	NA	NA	7.56	4.63
14	NA	NA	8.43	5.17
15	NA	NA	5.22	2.66
16	NA	NA	5.42	3.17
17	NA	NA	6.37	4.07
18	NA	NA	7.9	4.79
19	NA	NA	6.04	3.63
20	NA	NA	5.78	3.64
21	NA	NA	7.84	5.22
22	NA	NA	5.53	2.74
23	NA	NA	6.06	3.63
24	NA	NA	9.26	5.53
25	NA	NA	9.87	8.51
26	NA	NA	8.16	5.93
27	NA	NA	4.86	3.4
28	NA	NA	4.75	3.6
29	NA	NA	5.75	3.34
30	NA	NA	7.49	5.01
31	NA	NA	7.06	4.84
32	NA	NA	8.29	6.92
33	NA	NA	6.49	4.55
34	NA	NA	11.95	8.16
35	NA	NA	10.85	7.82
36	NA	NA	4.19	3.03
37	NA	NA	6.66	3.83
38	NA	NA	6.16	4.03
39	NA	NA	3.96	2.83
40	NA	NA	5.06	2.53
41	NA	NA	7.16	4.63
42	NA	NA	7.86	4.83
43	NA	NA	10.16	8.03
44	NA	NA	4.89	2.07
45	NA	NA	6.11	3.99
46	NA	NA	5.99	3.48
47	NA	NA	5.91	3.37
48	NA	NA	11.85	8.91
49	NA	NA	6.74	4.44
50	NA	NA	4.92	2.51
51	NA	NA	6.62	3.27

52	NA	NA	10.96	8.53
53	NA	NA	5.46	3.43
54	NA	NA	8.76	6.13
55	NA	NA	8.76	4.83
56	NA	NA	5.56	3.43
57	NA	NA	7	4.91
58	NA	NA	7.16	3.93
59	NA	NA	6.76	4.53
60	NA	NA	5.66	2.83
61	NA	NA	3.96	1.33
62	NA	NA	5.06	2.73
63	NA	NA	5.66	3.53
64	NA	NA	7.65	4.91
65	NA	NA	6.6	4.81
66	NA	NA	6.55	4.41
67	NA	NA	5.04	2.33
68	NA	NA	9.39	7.07
69	NA	NA	6.19	4.79
70	NA	NA	5.86	3.33
71	NA	NA	5.56	3.63
72	NA	NA	5.56	2.93
73	NA	NA	6.86	3.73
74	NA	NA	6.18	4.41
75	NA	NA	7.76	4.93
76	NA	NA	8.16	4.33
77	NA	NA	8.54	6.27
78	NA	NA	11.52	9.22
79	NA	NA	9.86	8.46
80	NA	NA	6.49	4.87
81	NA	NA	6.45	4.32
82	NA	NA	7.22	5.57
83	NA	NA	6	4.38
84	NA	NA	7.24	3.83
85	NA	NA	5.78	4.88
86	NA	NA	7.31	5.65
87	NA	NA	11.49	8.21
88	NA	NA	9.02	7.22
89	NA	NA	6.25	4.56
90	NA	NA	7.32	5.32
91	NA	NA	13.72	9.52
92	NA	NA	8.12	5.82
93	NA	NA	4.61	3.21
94	NA	NA	8.92	6.82
95	NA	NA	8.41	5.46
96	NA	NA	7.42	4.92
97	NA	NA	11.42	7.92
98	NA	NA	5.92	3.42
99	NA	NA	6.82	5.22
100	NA	NA	8.52	5.12
101	NA	NA	5.88	4.26
102	NA	NA	9.02	6.1
103	NA	NA	9.52	7.12
104	NA	NA	7.44	5.28
105	NA	NA	7.82	5.92

106	NA	NA	6.93	4.13
107	NA	NA	7.02	4.62

Lymphocytes(x109)	Monocytes(x109)	Eosinophils(x109)	Basophils(x109)	NLR
1.54	0.54	0.16	0.05	2.11039
1.15	0.56	0.13	0.05	4.286957
1.36	0.81	0.2	0.03	3.639706
1.76	0.57	0.2	0	2.630682
2.36	0.37	0.1	0	1.241525
1.04	0.21	0.13	0.02	3.596154
1.46	0.47	0.2	0	2.486301
0.94	0.33	0.06	0.01	3.617021
2.13	0.53	0.04	0.02	1.215962
2.86	0.57	0.1	0	1.863636
1.76	0.47	0.2	0	2.005682
1.66	0.47	0.1	0	5.5
2.26	0.57	0.1	0	2.048673
2.29	0.58	0.36	0.03	2.257642
1.88	0.48	0.18	0.02	1.414894
1.57	0.41	0.26	0.01	2.019108
1.73	0.51	0.04	0.02	2.352601
2.11	0.77	0.16	0.07	2.270142
1.76	0.41	0.22	0.02	2.0625
1.46	0.52	0.13	0.03	2.493151
1.72	0.74	0.14	0.02	3.034884
2.2	0.43	0.13	0.03	1.245455
1.76	0.47	0.2	0	2.0625
2.66	0.87	0.2	0	2.078947
0.92	0.39	0.01	0.04	9.25
1.46	0.67	0.1	0	4.061644
1.1	0.31	0.03	0.02	3.090909
0.93	0.18	0.02	0.02	3.870968
1.94	0.34	0.11	0.02	1.721649
1.71	0.57	0.17	0.03	2.929825
1.23	0.72	0.24	0.03	3.934959
0.79	0.52	0.06	0	8.759494
1.68	0.21	0.04	0.01	2.708333
2.79	0.96	0	0.04	2.924731
2.17	0.63	0.19	0.04	3.603687
0.82	0.25	0.08	0.01	3.695122
1.96	0.67	0.1	0.1	1.954082
1.56	0.57	0	0	2.583333
0.86	0.27	0	0	3.290698
1.96	0.47	0.1	0	1.290816
1.66	0.77	0.1	0	2.789157
2.26	0.67	0.1	0	2.137168
1.16	0.67	0.3	0	6.922414
2.32	0.45	0.04	0.01	0.892241
1.45	0.56	0.07	0.04	2.751724
1.84	0.5	0.15	0.02	1.891304
1.96	0.35	0.19	0.04	1.719388
2.33	0.48	0.12	0.01	3.824034
1.74	0.48	0.05	0.03	2.551724
1.85	0.4	0.13	0.03	1.356757
2.58	0.53	0.19	0.05	1.267442

1.96	0.37	0.1	0	4.352041
1.46	0.47	0.1	0	2.349315
1.76	0.77	0.1	0	3.482955
2.56	0.57	0.8	0	1.886719
1.46	0.57	0.1	0	2.349315
1.67	0.32	0.07	0.03	2.94012
2.16	0.67	0.3	0.1	1.819444
1.66	0.47	0.1	0	2.728916
2.26	0.47	0.1	0	1.252212
2.06	0.47	0.1	0	0.645631
1.66	0.57	0.1	0	1.644578
1.46	0.57	0.1	0	2.417808
2.02	0.45	0.26	0.01	2.430693
1.28	0.41	0.08	0.02	3.757813
1.5	0.52	0.08	0.04	2.94
2.13	0.46	0.1	0.02	1.093897
1.76	0.42	0.11	0.03	4.017045
0.86	0.36	0.15	0.03	5.569767
1.66	0.47	0.4	0	2.006024
1.26	0.47	0.2	0	2.880952
1.76	0.67	0.2	0	1.664773
2.26	0.47	0.4	0	1.650442
0.99	0.57	0.18	0.03	4.454545
1.96	0.77	0.1	0	2.515306
2.56	0.77	0.4	0.1	1.691406
1.58	0.55	0.12	0.02	3.968354
1.5	0.6	0.2	0	6.146667
1	0.28	0.11	0.01	8.46
0.99	0.49	0.12	0.02	4.919192
1.6	0.35	0.15	0.03	2.7
1.01	0.31	0.31	0.02	5.514851
1.08	0.45	0.06	0.03	4.055556
2.56	0.71	0.1	0.04	1.496094
0.46	0.35	0.08	0.01	10.6087
0.99	0.59	0.07	0.01	5.707071
2.52	0.68	0.04	0.04	3.257937
1.4	0.4	0	0	5.157143
1.1	0.45	0.1	0.04	4.145455
1.6	0.4	0	0	3.325
2.9	1.3	0	0	3.282759
1.7	0.5	0.1	0	3.423529
1.09	0.2	0.09	0.02	2.944954
0.9	0.8	0.4	0	7.577778
2.14	0.69	0.11	0.01	2.551402
1.9	0.5	0.1	0	2.589474
2.2	0.7	0.6	0	3.6
2	0.4	0.1	0	1.71
0.9	0.5	0.2	0	5.8
2.6	0.5	0.3	0	1.969231
1.14	0.41	0.05	0.02	3.736842
1.95	0.68	0.25	0.04	3.128205
1.8	0.5	0.1	0	3.955556
1.53	0.41	0.2	0.02	3.45098
1.4	0.4	0.1	0	4.228571

1.9	0.4	0.47	0.03	2.173684
1.9	0.5	0	0	2.431579

LMR	NIHSS	mRS	Infarct volume	Gender	Age	Hypertension
2.851852	7	3	1.68	Male	61	Yes
2.053571	4	2	1.52	Male	60	Yes
1.679012	3	1	3.48	Male	53	Yes
3.087719	3	1	1.36	Male	77	Yes
6.378378	5	2	2.36	Female	61	Yes
4.952381	5	3	8.3	Male	67	Yes
3.106383	3	1	1.28	Male	64	Yes
2.848485	6	2	2.54	Male	60	Yes
4.018868	4	2	1.8	Male	60	No
5.017544	6	3	2.16	Male	54	Yes
3.744681	3	0	1.1	Male	72	Yes
3.531915	3	0	1.16	Female	47	Yes
3.964912	5	2	1.04	Male	50	No
3.948276	3	1	1.16	Male	58	Yes
3.916667	5	3	1.52	Male	55	Yes
3.829268	6	3	1.3	Male	63	Yes
3.392157	4	2	1.42	Female	62	No
2.74026	8	3	2.14	Male	64	Yes
4.292683	6	2	1.96	Male	47	Yes
2.807692	4	1	2.14	Male	55	Yes
2.324324	3	1	1.16	Male	46	Yes
5.116279	6	3	1.28	Female	73	Yes
3.744681	4	2	3.1	Female	54	Yes
3.057471	2	0	2.36	Male	58	No
2.358974	8	3	1.9	Female	71	Yes
2.179104	9	3	3.46	Female	80	No
3.548387	5	1	1.8	Female	71	Yes
5.166667	6	2	6.6	Female	54	No
5.705882	4	1	1.22	Male	62	Yes
3	3	0	1.12	Male	59	No
1.708333	4	1	1.76	Male	74	Yes
1.519231	5	2	2.22	Male	57	Yes
8	3	0	1.3	Female	63	Yes
2.90625	5	1	2.3	Male	42	Yes
3.444444	9	3	5.54	Female	61	Yes
3.28	4	1	1.44	Male	88	No
2.925373	3	0	1.32	Male	52	No
2.736842	5	1	13.95	Male	59	No
3.185185	4	1	1.76	Female	61	No
4.170213	5	2	1.26	Female	53	No
2.155844	7	3	1.44	Male	39	No
3.373134	10	3	2.72	Male	46	Yes
1.731343	7	2	14.97	Male	47	No
5.155556	4	1	26.8	Female	57	Yes
2.589286	6	2	1.56	Female	56	No
3.68	3	0	1.64	Male	69	No
5.6	3	0	1.46	Male	59	No
4.854167	4	1	2.86	Male	26	No
3.625	8	3	1.3	Male	52	No
4.625	3	1	1.5	Female	52	No
4.867925	4	1	2.12	Male	48	No

5.297297	4	1	8.76	Female	59	Yes
3.106383	5	2	1.94	Female	64	Yes
2.285714	3	0	1.92	Female	74	Yes
4.491228	5	1	1.32	Female	59	Yes
2.561404	6	2	1.72	Male	68	No
5.21875	4	1	3.24	Male	64	No
3.223881	4	0	2.22	Male	51	No
3.531915	5	2	1.54	Male	69	No
4.808511	4	2	3.74	Male	64	Yes
4.382979	3	0	1.72	Female	57	Yes
2.912281	5	2	1.6	Female	64	Yes
2.561404	6	3	4.94	Male	58	No
4.488889	8	3	1.66	Male	63	Yes
3.121951	4	1	4.12	Female	48	No
2.884615	3	0	2.72	Female	53	Yes
4.630435	3	0	1.3	Female	68	Yes
4.190476	4	2	3	Female	58	Yes
2.388889	5	2	1.36	Female	75	Yes
3.531915	3	1	24.5	Male	59	Yes
2.680851	2	0	1.26	Male	72	Yes
2.626866	4	1	10.68	Male	75	No
4.808511	6	2	1.66	Female	63	No
1.736842	5	2	1.24	Male	44	Yes
2.545455	5	2	1.7	Male	57	No
3.324675	2	0	1.34	Male	48	Yes
2.872727	5	4	7.54	Male	46	Yes
2.5	5	4	8	Male	59	No
3.571429	10	5	7.24	Male	72	Yes
2.020408	6	5	1	Female	54	No
4.571429	4	4	1.68	Female	51	Yes
3.258065	10	5	3.24	Male	81	Yes
2.4	8	4	3.12	Male	85	Yes
3.605634	22	6	1.74	Male	82	No
1.314286	15	6	94.38	Male	84	Yes
1.677966	26	5	112.16	Male	61	Yes
3.705882	21	6	4.64	Female	74	Yes
3.5	23	6	7.44	Female	86	Yes
2.444444	9	4	2.02	Male	61	No
4	14	5	98.84	Female	87	Yes
2.230769	12	5	2.64	Female	70	Yes
3.4	26	6	28.92	Female	69	No
5.45	16	5	24.78	Male	80	No
1.125	8	4	1.72	Male	77	No
3.101449	12	5	2.28	Male	76	No
3.8	8	4	5.64	Female	78	No
3.142857	7	4	1.6	Male	44	Yes
5	5	5	1.78	Male	53	Yes
1.8	10	5	13.94	Male	82	No
5.2	7	4	1.7	Male	44	Yes
2.780488	9	5	6.52	Male	55	Yes
2.867647	10	5	2.1	Male	63	Yes
3.6	8	4	18.04	Female	52	Yes
3.731707	9	4	6.86	Male	57	No
3.5	10	5	2.28	Female	58	No

4.75	9	4	5.6	Female	58	Yes
3.8	9	4	13.2	Male	57	No

Admission SP	Admission DP	Coronary heart disease	Atrial fibrillation	Diabetes
180	90	No	No	No
168	102	No	No	No
175	96	No	Yes	No
185	105	No	Yes	No
200	110	Yes	No	No
180	80	No	Yes	No
210	100	No	No	No
150	106	No	Yes	No
180	106	No	No	No
170	120	No	No	No
144	102	No	No	No
158	108	No	No	No
130	80	No	No	No
130	80	No	No	No
130	90	No	No	No
160	90	No	No	No
160	100	No	No	No
130	70	No	No	No
135	83	No	No	No
200	110	No	No	No
180	70	No	No	No
167	90	No	Yes	No
148	90	No	No	No
150	80	No	Yes	No
166	90	No	No	No
120	82	No	No	No
146	82	No	No	No
136	76	Yes	Yes	No
126	85	No	No	No
160	105	No	No	No
146	69	No	No	No
209	110	No	No	No
168	94	Yes	No	No
209	146	No	No	No
136	70	No	No	No
131	62	No	No	No
151	78	No	No	Yes
137	77	Yes	No	No
136	82	No	No	No
128	70	No	No	No
126	94	No	No	No
126	80	No	No	No
110	67	No	No	Yes
155	100	No	No	No
120	86	No	No	No
130	80	No	No	No
140	80	No	No	No
120	80	No	No	No
150	120	No	No	No
180	89	No	No	No
110	80	No	No	No

162	75	No	Yes	No
126	72	No	No	No
220	90	Yes	No	No
124	80	No	No	Yes
160	108	No	No	No
220	128	No	No	No
160	100	No	No	No
138	80	Yes	No	No
140	80	No	No	Yes
130	80	Yes	No	No
146	90	No	No	No
160	90	No	No	No
130	80	No	No	No
172	88	No	No	No
160	100	No	No	No
155	90	Yes	Yes	No
165	90	No	No	No
150	80	No	No	No
150	100	No	No	No
143	70	No	No	No
150	80	Yes	Yes	No
140	95	No	No	No
160	100	No	No	No
158	90	No	No	No
136	96	No	No	No
130	80	No	Yes	No
160	100	No	No	No
160	105	No	No	Yes
170	100	No	No	No
168	100	No	No	No
145	75	No	Yes	No
164	61	Yes	No	No
169	81	No	No	No
130	92	Yes	No	Yes
160	120	Yes	Yes	No
202	120	Yes	No	Yes
196	88	No	Yes	No
180	100	No	No	Yes
160	110	Yes	Yes	No
110	80	Yes	No	No
197	97	No	No	No
163	96	Yes	Yes	Yes
149	77	Yes	No	No
204	96	No	No	Yes
166	78	No	No	No
177	120	No	No	No
153	84	Yes	No	Yes
165	80	No	Yes	No
140	101	No	No	No
130	88	Yes	Yes	No
140	88	No	No	No
174	102	No	No	No
136	78	No	Yes	No
146	86	No	No	No

160	100	No	No	No
160	100	No	No	No

Hyperlipidemia	Previous stroke	Fasting blood glucose	TC	TG	LDL
No	Yes	4.8	3.9	1.44	2.22
No	No	5.81	4.1	1.26	2.7
Yes	No	6.63	5.6	4.25	2.89
No	No	5.32	4.76	0.8	2.99
No	No	4.33	4.25	1	2.27
Yes	No	4.9	5.73	1.53	4.01
No	No	3.92	4.18	0.77	2.64
Yes	No	5.7	4.59	2.82	2.97
No	Yes	9.05	4.43	0.93	2.64
Yes	No	4.22	4.43	2.36	2.81
Yes	Yes	13.03	5.02	1.86	3.38
No	No	5.9	4.66	1.37	2.85
Yes	No	6.92	5.54	1.61	4.2
Yes	No	4.98	5.17	2.26	2.99
Yes	No	5.1	5.06	2.47	3.09
No	Yes	5.2	3.55	0.87	1.85
Yes	No	7.4	5.32	1.68	3.16
No	No	5.67	4.99	1.4	2.27
Yes	Yes	5.91	5.08	3.27	3.16
No	Yes	4.64	4.52	1.03	3.07
Yes	Yes	4.04	4.5	3.57	2.79
Yes	No	6.8	3.73	3.02	1.57
No	No	5.15	4.33	0.64	3.26
No	No	5.25	4.6	1.67	3.25
Yes	Yes	4.44	4.27	1.88	2.68
Yes	No	5.15	5.33	4.44	2.47
Yes	No	4.62	5.34	0.95	3.52
No	No	4.22	4.77	1.16	2.98
Yes	No	5.48	3.69	2.14	1.98
No	No	4.99	4.2	1.05	2.72
No	No	6.2	3.64	0.67	1.98
Yes	No	5.29	5.66	0.89	3.24
No	No	9.14	4.73	1.04	3.23
Yes	No	5.41	4.97	1.41	3.68
No	No	10.04	3.67	1.33	2.37
No	No	5.97	2.95	1.3	1.53
Yes	No	12.44	5.36	2.22	3.78
No	No	13.66	3.58	0.88	2.61
No	No	5.69	4.75	0.76	2.89
No	No	4.53	4.22	0.79	2.6
No	No	7.55	5.07	1.46	3.12
No	No	13.69	4.01	0.59	2.37
No	No	4.89	3.89	0.68	2.75
No	No	5.71	4.48	1.54	2.79
No	No	5.83	3.42	1.19	1.82
No	No	5.11	4.21	1.16	3
Yes	No	4.23	4.29	1.97	2.83
Yes	No	4.78	5.33	1.64	3.75
No	No	5.35	4.62	0.86	2.72
Yes	No	3.82	6.35	1.14	4.48
Yes	No	4.66	4.05	2.18	2.09

No	No	5.07	4.62	0.52	2.57
No	Yes	4.99	3.04	0.73	1.45
Yes	No	6.34	5.78	1.01	3.92
Yes	No	5.43	4.05	2.38	2.51
Yes	No	4.51	3.71	2.09	2.28
No	No	6.33	3.83	1.21	2.26
Yes	No	5.23	3.98	1.82	2.54
No	No	4.89	3.58	1.5	2.1
No	Yes	4.03	4.48	1.9	2.3
No	No	4.73	3.9	1.56	2.39
Yes	Yes	4.55	5.58	1.53	3.59
Yes	No	4.97	4.23	3.02	2.44
Yes	No	4.78	3.91	2.6	2.91
Yes	No	4.57	5.59	1.14	3.27
Yes	No	5.85	5.95	1.2	4.65
Yes	No	6.69	4.34	1.89	2.23
No	No	4.94	4.71	0.92	2.55
Yes	No	4.93	4.12	2.03	2.08
No	No	4.99	4.44	1.22	3.06
No	No	7.98	4.75	1.15	3.32
No	No	3.98	4.28	1.07	2.63
No	No	4.61	3.26	2.57	1.52
Yes	No	6.27	6.66	2.66	4.51
No	No	7.55	3.72	0.88	2.1
No	No	4.78	3.64	1.12	2.33
No	No	5.46	3.61	1.65	2.37
Yes	No	4.75	6.18	1.84	4.21
Yes	No	8.09	2.84	0.77	2.05
Yes	No	5.06	6.06	1.4	3.5
No	No	4.81	3.79	1.42	2.43
No	Yes	5.59	4.62	1.36	2.81
Yes	No	11.08	3.1	0.52	1.8
No	No	4.98	4.38	0.57	2.73
No	No	5.09	2.67	0.8	1.48
No	Yes	4.47	3.89	1.15	2.48
No	Yes	6.02	5.01	1.18	3.25
No	No	5.28	4.49	0.96	3.03
No	No	8.76	3.73	0.78	1.97
No	No	10.23	4.41	1.07	2.72
Yes	No	5.69	4.56	0.88	3.44
No	No	6.59	5.89	2.59	3.61
No	Yes	5.2	4.15	0.56	2.89
No	Yes	5.67	4.48	1.54	2.78
Yes	No	5.77	6.17	2.5	4.08
No	No	5.38	3.87	1.51	2.47
No	No	5.49	3.98	1.21	2.61
No	No	10.38	3.51	1.37	2.11
No	No	5.05	3.87	1.44	2.6
Yes	No	4.61	3.41	3.48	1.61
Yes	No	11.63	6.77	1.65	4.84
No	No	5.7	4.32	1.09	2.6
No	No	7.41	4.75	1.07	3.28
No	No	6.42	4.19	1.56	2.72
Yes	No	4.98	5.13	2.48	2.98

No	No	7.49	4.66	1.41	2.89
Yes	No	3.99	4.13	2.5	2.11

HDL	Smoking	Alcohol drinking	Prior anti-platelets	Prior statins
1.07	Yes	Yes	No	No
0.99	Yes	Yes	No	No
1.41	Yes	Yes	No	No
1.26	No	No	No	No
1.28	No	No	No	No
1.14	Yes	Yes	No	No
1.3	No	No	Yes	Yes
0.89	Yes	Yes	No	No
1.24	Yes	Yes	Yes	Yes
1.1	Yes	Yes	No	No
1.04	No	No	Yes	Yes
1.3	No	No	No	No
1.03	Yes	Yes	No	No
1.08	Yes	Yes	No	No
1.13	Yes	Yes	No	No
1.12	Yes	No	Yes	Yes
1.26	No	No	No	No
1.81	Yes	Yes	No	No
0.87	Yes	Yes	No	No
1.14	Yes	Yes	Yes	Yes
1.06	Yes	No	Yes	No
0.8	No	No	Yes	Yes
1.04	No	No	No	No
0.84	Yes	No	No	No
0.91	No	No	No	No
0.92	No	No	No	No
1.16	No	No	No	No
1.03	No	No	No	No
0.81	Yes	Yes	No	No
0.82	Yes	Yes	No	No
1.11	Yes	No	No	Yes
1.4	Yes	Yes	No	No
1.16	No	No	Yes	No
1.16	Yes	Yes	No	No
0.91	No	No	No	No
1.03	No	No	No	No
0.94	Yes	Yes	No	No
0.7	Yes	Yes	No	No
1.13	No	No	No	No
1.13	No	No	No	No
0.97	No	Yes	No	No
1.32	Yes	Yes	No	No
0.97	Yes	No	No	No
1.05	No	No	No	No
1.07	No	No	No	No
0.92	Yes	No	No	No
0.77	Yes	Yes	No	No
0.91	Yes	No	No	No
1.14	No	No	No	No
0.94	No	No	No	No
1.06	Yes	Yes	No	No

1.43	No	No	No	No
1.1	No	No	Yes	Yes
1.18	No	No	No	No
1.05	No	No	No	No
0.99	Yes	Yes	No	No
1.26	Yes	Yes	No	No
0.98	No	No	No	No
0.83	No	No	No	No
1.12	No	No	No	No
0.91	No	No	No	No
1.22	No	No	Yes	Yes
0.9	Yes	Yes	No	No
0.9	No	No	No	No
1.73	No	No	No	No
1.01	No	No	No	No
0.89	No	No	No	No
1.39	No	No	No	No
1.14	No	No	No	No
0.9	Yes	Yes	No	No
0.96	Yes	Yes	No	No
1.2	Yes	Yes	No	No
0.72	No	No	No	No
0.94	No	No	No	No
1.03	Yes	No	No	No
0.94	No	No	No	No
0.83	Yes	No	No	No
1.23	Yes	Yes	No	No
0.54	No	No	No	Yes
1.31	No	No	No	No
0.88	No	No	No	No
1.02	No	No	No	No
1.07	No	Yes	No	No
1.16	Yes	No	No	No
0.87	No	No	Yes	No
0.93	Yes	No	No	No
1.12	No	No	No	No
1.28	No	No	No	No
1.13	No	Yes	No	No
1.37	No	No	No	No
1.07	No	No	No	No
1.34	No	No	No	No
1.14	No	No	No	No
1.07	Yes	Yes	No	No
1.02	Yes	No	No	No
1.03	No	No	No	No
0.96	Yes	Yes	No	No
0.88	Yes	Yes	No	No
0.69	Yes	No	No	No
0.73	Yes	No	No	No
1.01	Yes	Yes	No	No
1.3	No	No	No	No
1.12	No	No	No	No
0.62	Yes	No	No	No
1.03	No	No	No	No

1.21	No	No	No	No
1.17	Yes	Yes	No	No

Infection while in hospital	ACS/PCS	TOAST
No	ACS	Small vessel
No	ACS	Small vessel
No	PCS	Cardioembolic
No	ACS	Cardioembolic
No	ACS	Small vessel
No	ACS	Undetermined
No	PCS	Small vessel
No	ACS	Cardioembolic
No	ACS	Large vessel
No	ACS	Small vessel
No	ACS	Small vessel
No	PCS	Small vessel
No	ACS	Undetermined
No	ACS	Small vessel
No	ACS	Large vessel
No	ACS	Small vessel
No	PCS	Small vessel
No	PCS	Large vessel
No	ACS	Small vessel
No	ACS	Small vessel
No	PCS	Small vessel
Yes	ACS	Cardioembolic
No	ACS	Large vessel
No	PCS	Cardioembolic
Yes	ACS	Large vessel
No	ACS	Large vessel
No	ACS	Large vessel
No	ACS	Cardioembolic
No	ACS	Small vessel
Yes	ACS	Small vessel
No	ACS	Small vessel
No	ACS	Small vessel
Yes	ACS	Large vessel
No	ACS	Small vessel
Yes	ACS	Large vessel
No	PCS	Small vessel
No	ACS	Large vessel
No	PCS	Large vessel
No	ACS	Small vessel
No	PCS	Small vessel
No	PCS	Undetermined
No	ACS	Large vessel
Yes	ACS	Large vessel
Yes	ACS	Large vessel
No	ACS	Undetermined
No	ACS	Small vessel
No	ACS	Small vessel
No	ACS	Large vessel
Yes	ACS	Other reasons
No	PCS	Small vessel
No	ACS	Large vessel

Yes	ACS	Cardioembolic
No	ACS	Small vessel
Yes	PCS	Large vessel
No	ACS	Small vessel
No	ACS	Small vessel
Yes	ACS	Small vessel
No	ACS	Small vessel
No	ACS	Undetermined
No	ACS	Small vessel
No	PCS	Small vessel
No	ACS	Large vessel
No	ACS	Undetermined
No	ACS	Large vessel
Yes	ACS	Large vessel
No	ACS	Large vessel
No	ACS	Cardioembolic
No	ACS	Small vessel
No	ACS	Small vessel
No	ACS	Large vessel
No	ACS	Small vessel
No	ACS	Cardioembolic
Yes	ACS	Small vessel
No	ACS	Small vessel
No	ACS	Small vessel
No	ACS	Small vessel
No	PCS	Undetermined
No	ACS	Large vessel
Yes	ACS	Large vessel
Yes	ACS	Small vessel
Yes	PCS	Small vessel
No	ACS	Undetermined
Yes	ACS	Large vessel
Yes	PCS	Large vessel
Yes	ACS	Large vessel
No	PCS	Undetermined
Yes	ACS	Large vessel
Yes	PCS	Cardioembolic
No	PCS	Small vessel
No	ACS	Cardioembolic
Yes	PCS	Large vessel
No	ACS	Large vessel
Yes	ACS	Cardioembolic
No	PCS	Large vessel
No	PCS	Large vessel
No	PCS	Small vessel
No	ACS	Other reasons
No	ACS	Small vessel
No	ACS	Cardioembolic
No	ACS	Large vessel
Yes	ACS	Cardioembolic
Yes	PCS	Small vessel
No	ACS	Large vessel
No	ACS	Cardioembolic
Yes	ACS	Small vessel

Yes	ACS	Undetermined
No	PCS	Large vessel

3).Raw data for patients in Beta-blocker group

Patient No.	Drug	Dosage	Leukocytes(x109)	Neutroplils(x109)
1	Metoprolol	25mg	6.06	3.46
2	Metoprolol	25mg	7.2	3.69
3	Metoprolol	25mg	8.1	6.89
4	Metoprolol	50mg	6.73	4.04
5	Metoprolol	100mg	7.4	3.99
6	Metoprolol	25mg	6.27	3.68
7	Bisoprolol	2.5mg	11.1	9.89
8	Metoprolol	12.5mg	9.27	6.03
9	Metoprolol	50mg	5.87	3.26
10	Bisoprolol	5mg	5.57	2.81
11	Metoprolol	25mg	5.5	2.69
12	Metoprolol	25mg	7.7	4.79
13	Metoprolol	50mg	9.7	6.49
14	Metoprolol	50mg	6.9	4.39
15	Metoprolol	50mg	8.8	5.59
16	Metoprolol	25mg	7.8	4.09
17	Metoprolol	50mg	7.87	3.76
18	Atenolol	100mg	5.08	3.09
19	Labetolol	400mg	8.82	6.88
20	Metoprolol	50mg	7.56	6.04
21	Metoprolol	50mg	6.8	4.29
22	Metoprolol	12.5mg	10.5	6.89
23	Metoprolol	25mg	6.86	4.28
24	Bisoprolol	7.5mg	7.7	4.56
25	Metoprolol	50mg	6	4.27
26	Metoprolol	12.5mg	5.2	3.49
27	Metoprolol	50mg	5.5	3.19
28	Metoprolol	25mg	5.78	2.92
29	Metoprolol	50mg	5.41	3.12
30	Metoprolol	50mg	5.84	3.6
31	Metoprolol	12.5mg	5.99	3.13
32	Metoprolol	25mg	7.9	5.39
33	Metoprolol	Unknown	6.6	4.59
34	Metoprolol	50mg	8.3	5.09
35	Bisoprolol	5mg	5.45	3.05
36	Bisoprolol	5mg	7.33	3.76
37	Metoprolol	25mg	5.8	3.79
38	Metoprolol	25mg	7.07	3.56
39	Metoprolol	50mg	5.7	3.09
40	Metoprolol	25mg	4.3	2.29
41	Metoprolol	12.5mg	4.89	2.16
42	Metoprolol	25mg	8.8	6.09
43	Bisoprolol	2.5mg	4.07	2.84
44	Bisoprolol	5mg	9.02	5.95
45	Metoprolol	25mg	6.26	3.89
46	Metoprolol	25mg	7.26	5.5
47	Bisoprolol	2.5mg	12.7	9.99
48	Bisoprolol	5mg	7.9	4.19
49	Metoprolol	50mg	7.7	4.89
50	Metoprolol	25mg	16.8	14.51
51	Metoprolol	25mg	9.04	4.39

52	Metoprolol	25mg	9.07	6.07
53	Metoprolol	50mg	8.07	4.97
54	Metoprolol	25mg	8.07	6.47
55	Metoprolol	50mg	7.61	4.87
56	Metoprolol	12.5mg	10.06	7.12
57	Metoprolol	50mg	11	9.26
58	Metoprolol	50mg	7.25	5.98
59	Metoprolol	25mg	10.49	8.1
60	Metoprolol	50mg	11.53	9.97
61	Bisoprolol	5mg	6.93	5.67
62	Metoprolol	25mg	9.77	5.87
63	Metoprolol	Unknown	7.27	3.87
64	Metoprolol	50mg	6.44	4.58
65	Metoprolol	50mg	13.67	9.06
66	Metoprolol	25mg	6.67	4.57
67	Metoprolol	50mg	6.87	5.07
68	Metoprolol	25mg	9.98	7.07
69	Bisoprolol	2.5mg	9.87	8.77

Lymphocytes(x109)	Monocytes(x109)	Eosinophils(x109)	Basophils(x109)	NLR
1.97	0.49	0.11	0.03	1.756345
2.56	0.55	0.3	0.1	1.441406
0.86	0.35	0	0	8.011628
1.98	0.49	0.18	0.04	2.040404
2.76	0.45	0.2	0	1.445652
1.89	0.42	0.25	0.03	1.94709
0.76	0.45	0	0	13.01316
2.49	0.57	0.17	0.01	2.421687
1.95	0.46	0.16	0.04	1.671795
1.98	0.54	0.2	0.04	1.419192
2.26	0.45	0.1	0	1.190265
2.16	0.65	0.1	0	2.217593
2.56	0.65	0	0	2.535156
1.46	0.85	0.2	0	3.006849
2.46	0.55	0.2	0	2.272358
2.26	0.65	0.8	0	1.809735
3.41	0.56	0.11	0.03	1.102639
1.5	0.29	0.18	0.02	2.06
1.33	0.5	0.1	0.01	5.172932
1.01	0.36	0.12	0.03	5.980198
1.96	0.55	0	0	2.188776
2.56	0.75	0.3	0	2.691406
1.7	0.63	0.2	0.05	2.517647
2.27	0.68	0.15	0.04	2.008811
1.25	0.33	0.11	0.04	3.416
1.06	0.65	0	0	3.292453
1.56	0.55	0.2	0	2.044872
2.46	0.3	0.08	0.02	1.186992
1.67	0.35	0.23	0.04	1.868263
1.48	0.5	0.23	0.03	2.432432
2.16	0.58	0.09	0.03	1.449074
1.76	0.55	0.2	0	3.0625
1.26	0.45	0.3	0	3.642857
2.46	0.55	0.2	0	2.069106
2	0.28	0.1	0.02	1.525
2.7	0.71	0.13	0.03	1.392593
1.46	0.45	0.1	0	2.59589
2.85	0.52	0.08	0.06	1.249123
1.96	0.45	0.2	0	1.576531
1.56	0.45	0	0	1.467949
1.83	0.62	0.25	0.03	1.180328
2.16	0.45	0.1	0	2.819444
0.87	0.31	0.04	0.01	3.264368
1.96	0.68	0.4	0.03	3.035714
1.61	0.56	0.17	0.03	2.416149
1.31	0.34	0.09	0.02	4.198473
1.86	0.55	0.3	0	5.370968
2.86	0.65	0.2	0	1.465035
1.96	0.55	0.3	0	2.494898
1.36	0.75	0.15	0.03	10.66912
4.09	0.42	0.13	0.01	1.07335

2.3	0.5	0.2	0	2.63913
2.5	0.6	0	0	1.988
1.4	0.2	0	0	4.621429
1.99	0.6	0.13	0.02	2.447236
2.14	0.68	0.09	0.03	3.327103
1.51	0.17	0.05	0.01	6.13245
0.92	0.23	0.1	0.02	6.5
1.27	0.82	0.28	0.02	6.377953
1.03	0.35	0.15	0.03	9.679612
0.78	0.36	0.11	0.01	7.269231
2.8	0.4	0.6	0.1	2.096429
2.9	0.4	0.1	0	1.334483
1.53	0.27	0.05	0.01	2.993464
3.51	0.95	0.12	0.03	2.581197
1.7	0.3	0.1	0	2.688235
1.4	0.3	0.1	0	3.621429
2.47	0.27	0.14	0.03	2.862348
0.7	0.3	0.1	0	12.52857

LMR	NIHSS	mRS	Infarct volume	Gender	Age	Hypertension
4.020408	4	1	1.76	Male	62	Yes
4.654545	5	3	2.2	Male	77	Yes
2.457143	2	0	3.4	Male	52	Yes
4.040816	3	1	1.28	Male	63	Yes
6.133333	4	2	2.28	Female	49	Yes
4.5	2	0	1.48	Male	71	Yes
1.688889	2	0	1.14	Male	83	Yes
4.368421	7	3	2.16	Male	71	No
4.23913	2	1	1.68	Female	76	No
3.666667	4	2	1.12	Female	80	Yes
5.022222	5	3	3.5	Female	81	Yes
3.323077	7	3	4.04	Female	73	Yes
3.938462	4	1	1.22	Male	50	Yes
1.717647	3	0	1.74	Female	77	Yes
4.472727	3	0	1.98	Male	67	Yes
3.476923	5	2	3	Male	37	Yes
6.089286	6	3	4.84	Male	53	Yes
5.172414	7	3	4.02	Male	54	Yes
2.66	5	2	4.34	Female	32	Yes
2.805556	4	1	1.36	Female	51	Yes
3.563636	9	3	1.72	Female	73	Yes
3.413333	6	2	1.5	Male	58	Yes
2.698413	4	1	2.14	Male	58	Yes
3.338235	4	1	1.4	Male	51	Yes
3.787879	4	1	1.06	Female	73	Yes
1.630769	4	2	1.12	Female	62	Yes
2.836364	5	2	2.52	Male	83	No
8.2	6	3	1.24	Female	55	Yes
4.771429	4	1	1.26	Male	62	Yes
2.96	4	0	1.16	Male	62	Yes
3.724138	4	1	1.3	Male	91	Yes
3.2	4	2	1.56	Male	50	Yes
2.8	6	3	1.7	Male	70	Yes
4.472727	4	1	1.32	Male	50	Yes
7.142857	11	3	2.44	Male	37	Yes
3.802817	3	0	1.48	Male	57	Yes
3.244444	4	2	1.26	Female	80	Yes
5.480769	3	1	1.24	Male	86	Yes
4.355556	5	2	1.94	Male	49	Yes
3.466667	6	3	3.88	Female	66	Yes
2.951613	6	2	1.62	Male	64	Yes
4.8	4	1	2.3	Female	80	Yes
2.806452	4	1	1.46	Male	64	Yes
2.882353	3	2	5.3	Male	55	Yes
2.875	4	2	6.24	Female	67	Yes
3.852941	6	3	1.84	Male	54	Yes
3.381818	6	3	1.96	Male	85	Yes
4.4	3	1	2.48	Male	53	Yes
3.563636	3	0	1.14	Male	40	Yes
1.813333	11	6	84.4	Female	79	No
9.738095	4	5	7.84	Male	54	Yes

4.6	2	4	30.9	Female	68	Yes
4.166667	7	5	32.28	Male	59	Yes
7	3	4	17.7	Female	83	Yes
3.316667	9	5	12.76	Female	77	Yes
3.147059	8	4	1.88	Female	73	Yes
8.882353	4	4	3.12	Female	79	No
4	7	4	5.56	Male	81	Yes
1.54878	14	5	36.1	Male	70	Yes
2.942857	38	6	81.08	Female	64	Yes
2.166667	24	6	162.43	Male	72	Yes
7	8	5	10.44	Male	44	Yes
7.25	6	4	0.26	Female	76	Yes
5.666667	10	5	7.08	Male	64	Yes
3.694737	13	6	57.4	Female	79	Yes
5.666667	20	5	51.3	Female	69	Yes
4.666667	6	4	11.56	Male	58	Yes
9.148148	9	5	31.78	Male	57	Yes
2.333333	8	4	5.6	Male	63	Yes

Admission SP	Admission DP	Coronary heart disease	Atrial fibrillation	Diabetes
130	80	No	Yes	No
120	70	Yes	Yes	No
190	120	Yes	No	No
135	90	No	Yes	No
140	70	No	No	No
143	90	No	Yes	No
140	70	No	Yes	No
135	80	Yes	Yes	No
140	80	No	Yes	No
170	80	No	Yes	No
138	84	Yes	No	No
138	85	Yes	Yes	No
120	80	Yes	No	No
152	64	Yes	No	No
130	70	Yes	No	No
168	118	No	No	No
146	86	No	Yes	No
160	78	No	No	No
170	119	No	Yes	No
170	80	No	Yes	Yes
120	70	Yes	Yes	No
200	120	Yes	No	Yes
155	105	Yes	Yes	No
199	100	No	No	Yes
174	102	No	No	No
137	72	No	No	Yes
140	70	Yes	No	No
170	107	Yes	No	No
150	70	No	No	No
164	96	Yes	No	No
150	90	Yes	No	No
130	90	No	No	No
150	80	Yes	No	Yes
166	110	No	Yes	Yes
207	144	No	No	No
152	90	No	No	No
134	83	No	No	No
135	95	No	No	No
130	87	Yes	No	No
150	90	Yes	No	Yes
145	100	No	No	No
150	80	Yes	No	No
146	78	Yes	No	No
160	100	Yes	No	Yes
150	100	Yes	No	No
143	103	Yes	Yes	No
140	81	No	Yes	Yes
128	70	No	No	Yes
148	87	Yes	No	Yes
140	73	Yes	Yes	No
146	96	Yes	Yes	No

170	110	Yes	Yes	No
166	96	Yes	Yes	No
150	84	Yes	No	No
150	89	Yes	No	Yes
220	100	Yes	Yes	Yes
140	70	Yes	No	Yes
170	89	Yes	Yes	No
132	76	Yes	Yes	Yes
158	116	Yes	No	No
133	72	No	Yes	No
178	110	No	No	Yes
128	82	No	No	Yes
138	73	Yes	Yes	No
152	72	Yes	Yes	No
124	67	Yes	Yes	Yes
151	83	Yes	Yes	Yes
139	83	No	No	No
148	85	Yes	Yes	Yes

Hyperlipidemia	Previous stroke	Fasting blood glucose	TC	TG	LDL
Yes	No	4.93	4.2	3.26	2.11
Yes	No	4	2.75	2.24	1.49
No	No	6.18	4.62	0.86	2.91
No	No	5.26	5.22	1.7	3.26
No	No	5.52	4.96	1.2	3.3
No	Yes	4.9	4.45	1.05	2.66
No	Yes	8.09	4.12	1.64	2.46
Yes	Yes	3.99	5.43	2.52	3.4
No	No	4.32	3.17	0.84	1.5
No	No	4.6	2.67	0.95	1.13
No	No	4.91	3.3	0.88	2.08
No	No	4.44	3.07	0.94	1.54
Yes	No	4.76	5.14	1.84	3.04
No	Yes	5.61	4.01	1.54	2.36
Yes	Yes	5.71	3.69	2.15	2.15
No	No	5.68	4.95	1.13	3.34
Yes	No	5.53	4.34	1.72	2.81
No	No	5.13	3.75	1.12	2.34
No	No	3.55	4.15	0.92	2.02
Yes	Yes	4.71	6.34	2.21	3.99
No	No	5.51	3.19	0.89	1.54
Yes	No	5.16	4.88	4	2.08
Yes	No	5.21	4.61	1.97	2.84
Yes	No	10.61	4.23	3.46	2.28
Yes	No	5.78	4.65	0.95	3.14
No	Yes	5.7	3.6	1.39	2.24
No	No	6.01	3.53	0.52	2.2
Yes	No	9.51	4.99	0.8	3.68
No	No	4.4	3.72	1.24	2.23
No	No	9.89	2.76	0.82	1.47
Yes	No	10.51	4.14	1.8	2.81
No	No	4.75	3.21	1.52	1.78
No	Yes	8.12	4.51	1.08	3.01
No	No	6.53	4.66	1.62	3.13
No	No	3.66	4.16	1.53	3.32
Yes	Yes	6.97	4.48	5.38	1.73
No	Yes	5.63	4.11	1.54	2.72
Yes	No	6.14	6.52	1.16	4.05
No	No	5.95	4.46	1.47	3.03
Yes	Yes	5.37	3.46	3.47	1.87
Yes	No	5.88	6.2	1.32	4.59
No	No	4.35	2.88	0.72	1.17
No	No	5.15	3.27	0.46	1.6
Yes	No	7.83	3.69	2.13	1.99
No	No	5.24	4.21	0.87	2.36
No	Yes	4.43	3.09	0.68	1.01
Yes	No	4.91	2.76	0.99	1.38
No	Yes	10.76	3.7	1.01	2.54
Yes	No	10.13	5.55	2.89	3.69
No	No	4.21	3.7	0.91	1.94
No	No	5.06	4.97	1.08	2.99

Yes	No	6.26	6.7	1.69	4.6
Yes	No	6.02	3.91	3.48	2.05
Yes	No	4.94	5.58	2.04	3.62
Yes	Yes	5.02	5.45	3.54	3.36
Yes	No	5.96	3.67	2.2	2.05
No	No	9.93	3.62	1.35	1.89
No	Yes	8.32	2.68	0.88	1.21
No	No	8.14	3.81	1.07	1.94
Yes	Yes	6.35	4.92	5.7	3.26
No	Yes	9.78	3.24	1.15	1.98
Yes	No	4.51	4.75	1.95	3.02
No	No	4.48	3.96	0.88	2.46
No	No	4.95	2.48	1.86	1.19
No	No	3.92	4.8	0.85	2.91
No	No	5.73	3.02	1.41	2.11
No	Yes	4.46	3.19	1	1.87
No	Yes	4.95	3.07	1.08	1.64
No	No	5.63	3.42	0.8	2

HDL	Smoking	Alcohol drinking	Prior anti-platelets	Prior statins
0.92	Yes	Yes	Yes	No
0.76	Yes	Yes	Yes	Yes
1.42	No	No	No	No
1.06	Yes	Yes	Yes	Yes
0.93	No	No	Yes	Yes
1.17	Yes	Yes	Yes	No
1.02	No	No	Yes	No
1.06	No	No	Yes	Yes
1.08	Yes	No	No	No
0.91	No	No	Yes	Yes
0.99	No	No	No	No
1.04	No	No	No	No
1.06	No	No	No	No
1.16	No	No	Yes	No
0.82	No	No	Yes	Yes
1.09	Yes	Yes	No	No
0.93	Yes	Yes	No	No
0.87	No	No	No	No
1.48	No	No	No	No
0.96	No	No	Yes	Yes
1.14	No	No	No	No
0.88	No	Yes	No	No
1.05	Yes	Yes	No	No
0.92	Yes	Yes	No	No
1.11	No	No	No	No
0.71	No	No	No	No
1.08	No	No	No	No
1.22	No	No	No	No
1.04	Yes	No	No	No
0.79	Yes	No	No	No
0.85	No	No	No	No
0.91	Yes	Yes	Yes	Yes
1.12	Yes	No	Yes	Yes
0.82	Yes	Yes	No	No
0.8	Yes	Yes	No	No
0.66	Yes	Yes	Yes	Yes
0.96	No	No	No	No
1.78	No	No	No	No
0.98	Yes	Yes	No	No
0.92	No	No	Yes	Yes
0.82	No	Yes	No	No
1.37	No	No	Yes	Yes
1.12	No	No	Yes	Yes
0.9	No	No	Yes	Yes
1.13	No	No	Yes	Yes
1.5	Yes	Yes	Yes	Yes
1.22	Yes	Yes	No	No
1.02	Yes	Yes	No	No
0.74	Yes	No	Yes	Yes
1.19	No	No	Yes	No
1.3	Yes	Yes	No	No

1.09	No	No	No	No
0.7	Yes	Yes	No	No
1.18	No	No	No	No
0.84	No	No	Yes	Yes
1.06	No	No	Yes	Yes
1.02	No	No	No	Yes
0.8	No	No	No	Yes
1.25	No	No	Yes	Yes
1.18	No	No	No	No
0.74	Yes	Yes	No	No
0.98	No	Yes	No	No
1.19	No	No	No	No
0.56	No	No	Yes	Yes
1.55	No	No	Yes	Yes
0.62	No	No	No	No
0.94	No	No	Yes	No
0.97	Yes	Yes	Yes	Yes
1.26	No	No	No	No

Infection while in hospital	ACS/PCS	TOAST
No	PCS	Undetermined
No	ACS	Cardioembolic
No	ACS	Small vessel
No	PCS	Cardioembolic
No	ACS	Small vessel
No	ACS	Cardioembolic
Yes	PCS	Cardioembolic
No	ACS	Cardioembolic
No	ACS	Cardioembolic
No	ACS	Cardioembolic
No	ACS	Undetermined
No	ACS	Cardioembolic
No	PCS	Small vessel
No	ACS	Other reasons
No	ACS	Small vessel
No	ACS	Small vessel
No	ACS	Cardioembolic
No	ACS	Small vessel
No	ACS	Cardioembolic
No	ACS	Cardioembolic
No	ACS	Cardioembolic
No	PCS	Small vessel
No	ACS	Undetermined
No	ACS	Small vessel
No	ACS	Small vessel
No	PCS	Large vessel
Yes	ACS	Small vessel
Yes	ACS	Small vessel
No	ACS	Small vessel
No	PCS	Small vessel
Yes	ACS	Undetermined
No	PCS	Small vessel
No	ACS	Small vessel
No	PCS	Cardioembolic
No	ACS	Small vessel
No	ACS	Small vessel
No	PCS	Small vessel
No	ACS	Large vessel
Yes	ACS	Small vessel
No	ACS	Large vessel
No	ACS	Large vessel
No	ACS	Small vessel
No	ACS	Small vessel
No	ACS	Undetermined
Yes	ACS	Large vessel
No	ACS	Cardioembolic
No	ACS	Small vessel
No	ACS	Large vessel
No	PCS	Small vessel
No	ACS	Cardioembolic
No	ACS	Cardioembolic

No	ACS	Cardioembolic
Yes	ACS	Cardioembolic
No	PCS	Large vessel
Yes	PCS	Large vessel
No	PCS	Small vessel
Yes	ACS	Large vessel
No	ACS	Cardioembolic
Yes	PCS	Cardioembolic
Yes	ACS	Undetermined
Yes	ACS	Cardioembolic
Yes	PCS	Large vessel
Yes	PCS	Undetermined
Yes	ACS	Cardioembolic
Yes	ACS	Cardioembolic
Yes	ACS	Large vessel
No	ACS	Cardioembolic
No	ACS	Large vessel
No	ACS	Small vessel

4).Raw data for patients in ACEI/ARB group

Patient No.	Drug	Dosage	Leukocytes(x109)	Neutroplils(x109)
1	Enalapril	10mg	9.05	6.52
2	Perindopril	4mg	6.75	5
3	Captopril	25mg	7.95	5.5
4	Enalapril	10mg	7.55	4.7
5	Enalapril	20mg	8.42	5.64
6	Captopril	25mg	10.67	8.94
7	Enalapril	10mg	6.25	4.2
8	Benazepril	10mg	9.55	8
9	Benazepril	10mg	7.72	5.03
10	Benazepril	10mg	7.25	5
11	Enalapril	Unknown	8.55	5.8
12	Enalapril	20mg	3.95	2.2
13	Enalapril	10mg	6.95	4.51
14	Captopril	50mg	5.65	2.9
15	Benazepril	10mg	6.19	4.76
16	Irbesartan	0.15g	6.62	4.71
17	Telmisartan	40mg	10.75	8.3
18	Valsartan	80mg	7.95	5.7
19	Valsartan	80mg	6.05	3.2
20	Valsartan	80mg	7.12	5.05
21	Valsartan	80mg	8.5	6.13
22	Valsartan	80mg	6.84	4.46
23	Irbesartan	0.15g	6.45	4.5
24	Irbesartan	0.15g	6.15	4.1
25	Losartan	50mg	6.75	4.6
26	Irbesartan	0.15g	7.75	6
27	Irbesartan	0.15g	5.65	4.2
28	Valsartan	80mg	7.55	4.8
29	Losartan	50mg	12.65	9.7
30	Valsartan	80mg	8.45	5.6
31	Irbesartan	0.15g	11.25	6.3
32	Irbesartan	0.15g	10.71	5.88
33	Enalapril	10mg	9.25	6.3
34	Irbesartan	0.15g	8.75	6.3
35	Irbesartan	0.15g	5.34	3.74
36	Irbesartan	0.15g	8.21	5.7
37	Irbesartan	0.15g	6.44	4.37
38	Irbesartan	0.15g	9.85	6.2
39	Irbesartan	0.15g	6.35	3.7
40	Valsartan	80mg	7.05	5.1
41	Enalapril	10mg	7.65	5.8
42	Benazepril	10mg	7.6	4.4
43	Benazepril	10mg	11.3	6.1
44	Enalapril	10mg	4.6	3.03
45	Benazepril	10mg	4.74	2.64
46	Irbesartan	Unknown	5.99	3.69
47	Irbesartan	0.15g	5.5	3.4
48	Valsartan	80mg	7.3	4.9
49	Candesartan	8mg	9.4	6
50	Valsartan	40mg	6.75	4.2
51	Irbesartan	0.15g	15	12.2

52	Valsartan	80mg	7.2	5.3
53	Valsartan	80mg	4.9	2.6
54	Irbesartan	0.15g	6.4	4.4
55	Valsartan	80mg	9.45	7.37
56	Valsartan	80mg	7.86	6.36

Lymphocytes(x109)	Monocytes(x109)	Eosinophils(x109)	Basophils(x109)	NLR
1.8	0.62	0.06	0.04	3.622222
1.05	0.5	0.1	0.1	4.761905
1.65	0.5	0.2	0	3.333333
2.15	0.5	0.2	0	2.186047
2.27	0.22	0.27	0.02	2.484581
1.12	0.55	0.02	0.04	7.982143
1.55	0.5	0	0	2.709677
1.05	0.4	0	0	7.619048
1.86	0.48	0.31	0.04	2.704301
1.55	0.5	0.1	0.1	3.225806
1.95	0.5	0.2	0	2.974359
1.15	0.3	0.2	0	1.913043
1.96	0.34	0.13	0.02	2.30102
2.25	0.4	0.1	0	1.288889
0.88	0.45	0.06	0.03	5.409091
1.39	0.37	0.14	0.02	3.388489
1.75	0.4	0.2	0	4.742857
1.15	0.8	0.2	0	4.956522
2.25	0.3	0.2	0	1.422222
1.35	0.67	0.03	0.03	3.740741
1.67	0.59	0.07	0.03	3.670659
1.83	0.43	0.08	0.04	2.437158
1.45	0.4	0	0	3.103448
0.95	0.4	0.6	0	4.315789
1.35	0.6	0.1	0	3.407407
1.35	0.4	0.1	0	4.444444
1.05	0.3	0.1	0	4
2.15	0.5	0.1	0	2.232558
1.85	1	0.2	0	5.243243
1.95	0.6	0.3	0	2.871795
4.05	0.7	0.2	0.1	1.555556
3.78	0.8	0.16	0.08	1.555556
1.85	0.7	0.3	0.1	3.405405
1.95	0.4	0	0	3.230769
1.3	0.24	0.04	0.03	2.876923
1.77	0.51	0.21	0.02	3.220339
1.63	0.31	0.09	0.04	2.680982
2.75	0.6	0.2	0	2.254545
1.85	0.5	0.2	0	2
1.35	0.4	0.1	0	3.777778
1.45	0.4	0	0	4
2.1	0.6	0.5	0.1	2.095238
4	0.78	0.3	0	1.525
1.13	0.38	0.03	0.02	2.681416
1	1.04	0.04	0	2.64
1.09	0.52	0.14	0.01	3.385321
1.7	0.3	0.1	0	2
1.5	0.6	0.2	0.1	3.266667
2.2	0.9	0.1	0.1	2.727273
1.93	0.52	0.06	0.03	2.176166
1.3	1.1	0.3	0	9.384615

1.3	0.4	0.1	0	4.076923
1.3	0.4	0.6	0.1	2
1.4	0.6	0	0	3.142857
1.09	0.72	0.22	0.05	6.761468
0.78	0.49	0.18	0.03	8.153846

LMR	NIHSS	mRS	Infarct volume	Gender	Age	Hypertension
2.903226	6	3	10.18	Male	57	Yes
2.1	5	2	4	Female	82	Yes
3.3	9	3	2.3	Male	70	Yes
4.3	7	2	1.3	Male	52	Yes
10.31818	7	2	6.26	Female	68	Yes
2.036364	7	3	3.68	Male	63	Yes
3.1	3	0	1.62	Female	46	Yes
2.625	4	1	1.44	Male	37	Yes
3.875	6	2	9.64	Male	53	Yes
3.1	5	1	1.4	Male	62	Yes
3.9	7	2	1.4	Male	47	Yes
3.833333	5	1	1.2	Male	85	Yes
5.764706	4	1	1.52	Male	48	Yes
5.625	8	3	2.14	Male	53	Yes
1.955556	5	2	1.62	Female	67	Yes
3.756757	5	1	0.86	Female	80	Yes
4.375	4	0	1.82	Male	58	Yes
1.4375	4	1	2.08	Female	56	Yes
7.5	11	3	1.28	Female	70	Yes
2.014925	5	2	2.32	Male	49	Yes
2.830508	3	1	3.68	Male	57	Yes
4.255814	4	1	3.62	Female	93	Yes
3.625	5	0	1.18	Male	50	Yes
2.375	7	2	1.68	Male	69	Yes
2.25	5	1	1.16	Female	74	Yes
3.375	4	0	1.12	Male	78	Yes
3.5	5	2	1.62	Male	79	Yes
4.3	6	2	1.6	Male	67	Yes
1.85	5	1	1.76	Male	49	Yes
3.25	7	2	1.74	Male	61	Yes
5.785714	4	0	1.72	Male	56	Yes
4.725	7	2	1.7	Male	55	Yes
2.642857	6	2	2.08	Male	63	Yes
4.875	4	3	7.62	Male	69	Yes
5.416667	5	1	1.36	Female	68	Yes
3.470588	6	2	1.7	Female	69	Yes
5.258065	5	0	1.5	Male	65	Yes
4.583333	3	3	3.88	Male	56	Yes
3.7	4	0	1.1	Male	54	Yes
3.375	5	2	3.68	Male	58	Yes
3.625	6	1	1.56	Female	50	Yes
3.5	6	4	1.5	Male	59	Yes
5.128205	4	6	39.82	Male	64	Yes
2.973684	9	5	12.68	Male	63	Yes
0.961538	8	4	22.72	Female	60	Yes
2.096154	13	5	6.88	Male	86	Yes
5.666667	6	4	1.44	Male	60	Yes
2.5	7	4	3.38	Male	63	Yes
2.444444	7	4	3.16	Female	74	Yes
3.711538	3	4	17.32	Male	53	Yes
1.181818	6	5	26.42	Male	58	Yes

3.25	6	5	19.58	Male	66	Yes
3.25	7	4	2.4	Male	61	Yes
2.333333	9	5	8.18	Male	51	Yes
1.513889	10	5	2.66	Female	83	Yes
1.591837	30	6	106.25	Female	83	Yes

Admission SP	Admission DP	Coronary heart disease	Atrial fibrillation	Diabetes
123	83	Yes	No	No
146	81	Yes	No	No
157	89	No	No	Yes
170	110	Yes	No	No
150	98	No	Yes	No
160	113	Yes	No	Yes
145	105	Yes	No	No
160	100	No	No	Yes
177	90	Yes	No	No
150	80	No	No	No
166	80	No	No	No
160	70	Yes	No	No
136	74	No	Yes	No
190	110	No	No	No
212	110	No	No	No
131	65	Yes	Yes	No
124	71	No	No	No
179	101	No	No	No
158	90	Yes	No	Yes
142	90	No	Yes	No
119	70	No	No	No
138	98	Yes	No	Yes
120	80	No	Yes	No
128	68	Yes	No	No
168	82	No	No	No
220	100	No	No	Yes
130	80	No	No	Yes
168	108	Yes	No	No
97	66	No	No	Yes
138	90	Yes	No	No
190	120	No	Yes	No
200	105	No	No	No
150	95	No	Yes	Yes
158	92	Yes	No	No
160	70	No	Yes	Yes
160	94	No	No	No
156	82	No	Yes	Yes
145	90	Yes	No	Yes
100	70	No	No	No
130	84	No	Yes	No
150	80	No	No	Yes
136	72	No	No	No
144	91	No	Yes	Yes
146	94	No	No	No
140	70	No	Yes	Yes
123	83	No	Yes	No
158	80	Yes	No	No
194	102	No	No	Yes
150	100	Yes	Yes	Yes
137	96	Yes	No	No
148	80	Yes	No	No

150	90	No	Yes	No
170	110	No	Yes	No
150	90	No	Yes	Yes
178	89	Yes	No	Yes
160	90	Yes	Yes	No

Hyperlipidemia	Previous stroke	Fasting blood glucose	TC	TG	LDL
No	No	4.11	4.88	1.66	2.9
No	No	5.39	3.88	0.8	2.36
No	No	8.6	4.05	1.14	2.03
No	No	4.45	4.22	1.06	2.47
No	No	4.43	3.91	1.13	2.48
Yes	Yes	11.77	3.98	2.3	2.11
No	No	5.87	3.86	1.18	2.48
Yes	No	6.73	5.82	1.92	4.5
Yes	No	5.44	5.23	1.27	3.77
No	No	5.14	4.4	0.96	2.77
Yes	No	4.48	4.87	2.3	3.2
No	No	4.68	3.45	0.7	2.16
Yes	Yes	5.23	4	1.38	2.16
No	No	10.41	4.16	1.24	2.66
No	No	4.91	4.75	1.4	2.75
No	Yes	6.02	3.47	1.23	2.02
Yes	No	4.08	4.49	2.97	2.75
No	No	4.05	3.69	1.03	2.35
Yes	Yes	9.04	4.14	1.93	2.45
Yes	No	5.21	7.09	2.07	5.14
Yes	No	5.96	4.91	1.18	3.64
No	No	4.82	3.84	0.73	2.04
Yes	No	6.07	4.9	1.25	3.57
Yes	Yes	4.94	4.14	2.49	2.49
Yes	Yes	4.79	4.09	2.69	2.35
No	No	5.06	4.42	1.14	2.64
Yes	Yes	4.81	4.98	2.08	3.16
Yes	No	4.73	5.06	3.48	2.99
Yes	No	5.15	4.51	3.99	2.44
No	Yes	4.34	2.33	0.96	1.38
No	Yes	6.24	4.15	1.54	2.89
Yes	No	5.37	6.47	2.64	3.82
No	No	4.28	3.28	0.58	2.01
No	No	5.61	4.89	1.09	3.29
No	No	4.01	4.76	1.41	2.79
Yes	Yes	6.89	5.55	2.66	3.38
No	No	4.37	4.59	0.86	2.72
No	Yes	4.39	4.15	0.5	2.53
Yes	No	4.46	4.09	1.33	2.49
Yes	No	6.42	3.65	2.45	2.13
No	No	12.09	3.82	1.55	2.31
Yes	Yes	4.71	6.93	4.48	4.62
Yes	Yes	6.79	4.69	2.25	2.83
Yes	No	6.23	5.63	1.86	3.59
No	Yes	11.02	4.72	1.34	3
No	Yes	4.08	2.42	0.6	1.36
No	No	5.23	2.68	1.24	0.81
Yes	No	5.4	5.38	1.64	3.71
No	No	4.88	4.91	1.29	3.1
Yes	No	6.08	5.69	8.27	2.04
No	Yes	4.38	2.63	1.03	1.26

No	Yes	5.5	3.25	1.01	1.68
Yes	No	6.71	4.94	2.09	3.33
Yes	Yes	5.77	4.36	1.84	2.91
Yes	No	10.2	5.86	1.02	4.55
No	Yes	5.71	3.3	1.06	1.65

HDL	Smoking	Alcohol drinking	Prior anti-platelets	Prior statins
1.25	Yes	Yes	No	No
1.05	No	No	No	No
1.1	No	No	No	No
1.45	Yes	Yes	No	No
0.93	No	No	Yes	No
0.66	Yes	No	Yes	Yes
1.14	No	No	No	No
1.04	No	No	No	No
0.84	No	No	No	No
1.21	Yes	Yes	No	No
0.86	Yes	Yes	No	No
0.95	No	No	No	No
1.27	Yes	Yes	No	No
0.84	Yes	Yes	No	No
1.02	No	No	No	No
0.93	No	No	No	No
0.9	Yes	No	No	No
0.88	No	No	No	No
1.03	No	No	No	No
1.11	Yes	Yes	No	No
0.94	No	No	No	No
1.28	No	No	No	No
1.03	Yes	No	No	No
0.89	Yes	Yes	Yes	Yes
0.79	No	No	No	No
1.16	No	No	No	No
0.9	No	No	Yes	Yes
0.79	No	No	No	No
0.79	Yes	Yes	No	No
0.62	No	No	No	No
0.93	Yes	Yes	No	No
0.99	Yes	Yes	No	No
1.06	Yes	Yes	No	No
1.34	Yes	No	No	No
1.23	No	No	No	No
0.98	No	No	Yes	No
1.49	Yes	Yes	No	No
1.32	Yes	No	No	No
0.92	Yes	Yes	No	No
1.03	No	No	No	No
1.02	No	No	No	No
0.92	Yes	Yes	No	No
1.26	No	No	No	No
1.06	No	Yes	No	No
1.04	No	No	No	No
0.92	No	No	Yes	Yes
1.53	Yes	Yes	No	No
1.18	Yes	Yes	No	No
1.08	No	No	Yes	Yes
0.8	Yes	Yes	No	No
1.04	No	No	Yes	Yes

0.97	No	Yes	No	No
0.97	No	Yes	No	No
0.82	Yes	No	Yes	Yes
1.36	No	No	Yes	Yes
1.19	No	No	Yes	Yes

Infection while in hospital	ACS/PCS	TOAST
No	PCS	Large vessel
No	ACS	Large vessel
No	ACS	Small vessel
Yes	PCS	Small vessel
No	ACS	Cardioembolic
No	ACS	Small vessel
Yes	ACS	Small vessel
No	PCS	Small vessel
No	ACS	Large vessel
No	PCS	Small vessel
No	ACS	Small vessel
Yes	ACS	Small vessel
No	ACS	Cardioembolic
No	PCS	Large vessel
No	ACS	Small vessel
No	ACS	Cardioembolic
No	ACS	Large vessel
No	ACS	Large vessel
Yes	PCS	Small vessel
No	ACS	Cardioembolic
Yes	ACS	Undetermined
No	PCS	Undetermined
Yes	PCS	Cardioembolic
No	ACS	Large vessel
Yes	ACS	Small vessel
No	PCS	Small vessel
No	ACS	Small vessel
No	PCS	Small vessel
Yes	ACS	Small vessel
No	ACS	Large vessel
No	ACS	Cardioembolic
Yes	ACS	Small vessel
No	ACS	Cardioembolic
No	PCS	Small vessel
Yes	PCS	Cardioembolic
No	PCS	Small vessel
No	ACS	Cardioembolic
No	ACS	Large vessel
No	PCS	Small vessel
No	ACS	Undetermined
No	ACS	Large vessel
Yes	ACS	Large vessel
No	PCS	Undetermined
Yes	ACS	Large vessel
No	ACS	Cardioembolic
Yes	ACS	Undetermined
Yes	ACS	Small vessel
Yes	ACS	Large vessel
No	PCS	Cardioembolic
Yes	PCS	Large vessel
No	ACS	Other reasons

Yes	PCS	Cardioembolic
No	ACS	Cardioembolic
Yes	ACS	Cardioembolic
Yes	PCS	Small vessel
No	ACS	Cardioembolic
