# Supplementary material for: Premorbid Use of Beta-Blockers or Angiotensin-Converting Enzyme Inhibitors/Angiotensin Receptor Blockers in Patients with Acute Ischemic Stroke
Source: Oxid Med Cell Longev. 2023 Feb 1;2023:7733857. doi: 10.1155/2023/7733857 (PMC9908343; doi:10.1155/2023/7733857)
Supplement: Supplementary Materials — Supplementary Table 1: baseline data for healthy controls and matched controls. Supplementary Table 2: dosages of different beta-blockers and ACEIs/ARBs used prior to stroke onset expressed as percentages of maximum recommended therapeutic dose. Supplementary File 1: raw data from patients in the healthy control group, the matched control group, the beta-blocker group, and the ACEI/ARB group. [file 7733857.f1.zip › Supplementary table 1 (1).docx]

**Supplementary table 1. Baseline data for healthy controls and matched controls**

| Characteristic and  Variables | Healthy controls  (n=45) | Matched controls  (n=107) |
| --- | --- | --- |
| Male，gender, n (%) | 24 (53.3) | 68 (63.6) |
| Age (years), mean ± SD | 60.3±12.2 | 61.4±11.6 |
| Hypertension, n (%) | 10 (22.2) | 64 (59.8) *** |
| Coronary heart disease, n (%) | 6 (13.3) | 19 (17.8) |
| Atrial fibrillation, n (%) | 1 (2.2) | 19 (17.8) * |
| Diabetes, n (%) | 3 (6.7) | 11 (10.3) |
| Hyperlipidemia, n (%) | 16 (35.6) | 45 (42.1) |
| Previous stroke, n (%) | 3 (6.7) | 16 (15.0) |
| Prior anti-platelets, n (%) | 1 (2.2) | 11 (10.3) |
| Prior statins, n (%) | 1 (2.2) | 10 (9.3) |
| Smoking, n (%) | 8 (17.8) | 49 (45.8) ** |
| Alcohol drinking, n (%) | 7 (15.6) | 37 (34.6) * |
| Infection while in hospital, n (%) | NA | 27 (25.2) |
| Admission systolic blood pressure, mean ± SD, mmHg | 135.4±20.4 | 155.2±24.6*** |
| Admission diastolic blood pressure, mean ± SD, mmHg | 79.4±10.9 | 90.7±14.8*** |
| Fasting blood glucose, mean ± SD, mmol/L | 5.17±1.71 | 6.02±2.10* |
| Admission blood lipid |  |  |
| TC, mean ± SD, mmol/L | 4.70±1.02 | 4.48±0.83 |
| TG, mean ± SD, mmol/L | 1.40±0.90 | 1.54±0.79 |
| LDL-C, mean ± SD, mmol/L | 2.86±0.78 | 2.79±0.70 |
| HDL, mean ± SD, mmol/L | 1.16±0.23 | 1.06±0.20* |
| Admission NIHSS, median (IQR) | NA | 5 (4-8) |
| Infarct volume, mL, median (IQR) | NA | 2.02 (1.50-4.64) |
| Stroke subtype (TOAST) |  |  |
| Large vessel, n (%) | NA | 35 (32.7) |
| Small vessel, n (%) | NA | 45 (42.1) |
| Cardioembolic, n (%) | NA | 15 (14.0) |
| Other reasons, n (%) | NA | 2 (1.9) |
| Undetermined, n (%) | NA | 10 (9.3) |
| Leukocytes (x10^9^) | 6.62±1.72 | 7.17±1.95 |
| Neutrophils (x10^9^) | 4.05±1.62 | 4.79±1.76* |
| Lymphocytes (x10^9^) | 1.91±0.63 | 1.70±0.53* |
| Monocytes (x10^9^) | 0.51±0.42 | 0.52±0.17 |
| Eosinophils (x10^9^) | 0.13±0.09 | 0.15±0.12 |
| Basophils (x10^9^) | 0.04±0.03 | 0.02±0.02 |
| NLR | 2.53±1.89 | 3.18±1.79* |
| LMR | 4.71±1.77 | 3.48±1.18*** |

**p*<0.05, Healthy controls versus Matched controls.

***p*<0.01, Healthy controls versus Matched controls.

*** *p*<0.001, Healthy controls versus Matched controls.

*SD*, standard deviation; *IQR*, interquartile range; *NA*, not applicable; *NIHSS*, National Institutes of Health Stroke Scale score; *TC*, total cholesterol; *TG*, triglyceride; *LDL*-*C*, low-density lipoprotein cholesterol; *HDL*, high-density lipoprotein cholesterol; *NLR*, neutrophil to lymphocyte ratio; *LMR*, lymphocyte to monocyte ratio.
