# Supplementary material for: Premorbid Use of Beta-Blockers or Angiotensin-Converting Enzyme Inhibitors/Angiotensin Receptor Blockers in Patients with Acute Ischemic Stroke
Source: Oxid Med Cell Longev. 2023 Feb 1;2023:7733857. doi: 10.1155/2023/7733857 (PMC9908343; doi:10.1155/2023/7733857)
Supplement: Supplementary Materials — Supplementary Table 1: baseline data for healthy controls and matched controls. Supplementary Table 2: dosages of different beta-blockers and ACEIs/ARBs used prior to stroke onset expressed as percentages of maximum recommended therapeutic dose. Supplementary File 1: raw data from patients in the healthy control group, the matched control group, the beta-blocker group, and the ACEI/ARB group. [file 7733857.f1.zip › Supplementary table 2 (1).docx]

**Supplementary table 2. Dosages of different beta-blockers and ACEIs / ARBs used prior to stroke onset expressed as percentages of maximum recommended therapeutic dose**

| Drugs | *n* | ≥25% MRTD (*n*) | ≥50% MRTD (*n*) | Dose unknown (*n*) |
| --- | --- | --- | --- | --- |
| Metoprolol | 56 | 48 | 24 | 2 |
| Bisoprolol | 11 | 11 | 7 | 0 |
| Atenolol | 1 | 1 | 1 | 0 |
| Labetalol | 1 | 1 | 1 | 0 |
| Enalapril | 10 | 9 | 2 | 1 |
| Benazepril | 7 | 7 | 0 | 0 |
| Perindopril | 1 | 1 | 1 | 0 |
| Captopril | 3 | 3 | 3 | 0 |
| Valsartan | 14 | 14 | 13 | 0 |
| Losartan | 2 | 2 | 2 | 0 |
| Irbesartan | 17 | 16 | 16 | 1 |
| Telmisartan | 1 | 1 | 1 | 0 |
| Candesartan | 1 | 1 | 1 | 0 |

MRTD: maximum recommended therapeutic dose.
